# Adaptive Differentiated Parrot Optimization: A Multi-Strategy Enhanced Algorithm for Global Optimization with Wind Power Forecasting Applications

**DOI:** 10.3390/biomimetics10080542

**Published:** 2025-08-18

**Authors:** Guanjun Lin, Mahmoud Abdel-salam, Gang Hu, Heming Jia

**Affiliations:** 1 School of Information Engineering, Sanming University, Sanming 365004, China; guanjun.lin@fjsmu.edu.cn; 2 Faculty of Computers and Information Science, Mansoura University, Mansoura 35516, Egypt; mahmoud20@mans.edu.eg; 3 Department of Applied Mathematics, Xi’an University of Technology, Xi’an 710054, China; hugang@xaut.edu.cn

**Keywords:** parrot optimization algorithm, energy forecasting, dimension learning-based hunting, LSTM

## Abstract

The Parrot Optimization Algorithm (PO) represents a contemporary nature-inspired metaheuristic technique formulated through observations of Pyrrhura Molinae parrot behavioral patterns. PO exhibits effective optimization capabilities by achieving equilibrium between exploration and exploitation phases through mimicking foraging behaviors and social interactions. Nevertheless, during iterative progression, the algorithm encounters significant obstacles in preserving population diversity and experiences declining search effectiveness, resulting in early convergence and diminished capacity to identify optimal solutions within intricate optimization landscapes. To overcome these constraints, this work presents the Adaptive Differentiated Parrot Optimization Algorithm (ADPO), which constitutes a substantial enhancement over baseline PO through the implementation of three innovative mechanisms: Mean Differential Variation (MDV), Dimension Learning-Based Hunting (DLH), and Enhanced Adaptive Mutualism (EAM). The MDV mechanism strengthens the exploration capabilities by implementing dual-phase mutation strategies that facilitate extensive search during initial iterations while promoting intensive exploitation near promising solutions during later phases. Additionally, the DLH mechanism prevents premature convergence by enabling dimension-wise adaptive learning from spatial neighbors, expanding search diversity while maintaining coordinated optimization behavior. Finally, the EAM mechanism replaces rigid cooperation with fitness-guided interactions using flexible reference solutions, ensuring optimal balance between intensification and diversification throughout the optimization process. Collectively, these mechanisms significantly improve the algorithm’s exploration, exploitation, and convergence capabilities. Furthermore, ADPO’s effectiveness was comprehensively assessed using benchmark functions from the CEC2017 and CEC2022 suites, comparing performance against 12 advanced algorithms. The results demonstrate ADPO’s exceptional convergence speed, search efficiency, and solution precision. Additionally, ADPO was applied to wind power forecasting through integration with Long Short-Term Memory (LSTM) networks, achieving remarkable improvements over conventional approaches in real-world renewable energy prediction scenarios. Specifically, ADPO outperformed competing algorithms across multiple evaluation metrics, achieving average R^2^ values of 0.9726 in testing phases with exceptional prediction stability. Moreover, ADPO obtained superior Friedman rankings across all comparative evaluations, with values ranging from 1.42 to 2.78, demonstrating clear superiority over classical, contemporary, and recent algorithms. These outcomes validate the proposed enhancements and establish ADPO’s robustness and effectiveness in addressing complex optimization challenges.

## 1. Introduction

An optimization search is a central problem in computational science, concerned with searching through combinations of variables that fulfill certain constraints, but in a way that minimizes or maximizes some objectives that have been pre-defined [1]. Classical optimization tools solve such multi-variable constraint situations by using strict mathematical developments based on the properties of problem-space structures [2]. They identify the best possible solutions in confined areas using direct or indirect computational processes that normally need accurate mathematical expressions of objective functions and boundary constraints, such as linear programming [3] and gradient techniques [4], as well as conjugate gradient algorithms. However, these traditional methods often fail to scale well for large, nonlinear, or high-dimensional spaces [5].

Due to the increasing complexity and diversity of optimization problems in fields such as trajectory optimization [6], cybersecurity systems [7], the design of engineering systems [8], machine learning prediction [9], economic dispatch in microgrids [10], and blockchain [11], there is a growing demand for flexible and robust solution methods. Algorithms of metaheuristics have emerged as efficient alternatives, employing computational intelligence and stochastic search techniques to provide high-precision solutions to complicated real-life optimization problems [12,13].

Various metaheuristic approaches (MAs), such as Genetic Algorithms (GAs) [14] and Differential Evolution (DE) [15], Particle Swarm Optimization (PSO) [16], the Gray Wolf Optimizer (GWO) [17], Ant Colony Optimization (ACO) [18], Gravitational Search Algorithm (GSA) [19], and various nature-inspired variants [20,21,22,23,24,25,26,27] have shown effectiveness but still face challenges like premature convergence and poor performance in complex landscapes. With the advances in contemporary science and technology, and the development of different domains, they present increasingly high-dimensional optimization problems with not only increasing complexity but also multimodality and many local optima [28,29].

Although these smart algorithms have proven to be better at handling real-world optimization problems, they are still limited by factors such as limited exploration abilities, performance slowdowns at high complexity, and slow convergence with more complicated and demanding problems. Moreover, the No Free Lunch (NFL) theorem shows that no algorithm can outperform all other algorithms in all possible optimization problems [30]. This principle highlights the critical importance of developing effective and fast practical applications. As a result, we can state that further improvement and better performance of these algorithms are essential in order to obtain more sensible and optimal solutions in practical optimization problems.

To deal with the inevitable weakness of algorithms, researchers often employ domain-specific solutions or combinations of several strategies to enhance performance. For example, Zou and Wang presented an Improved Multi-Strategy Beluga Whale Optimization (IMS-BWO) algorithm that enhances the original BWO through three key strategies—roulette-based fitness distance balancing, random differential restart, and non-monopoly search with Levy motion—achieving superior performance with 69.8–100% win rates against competing algorithms [31]. Fakhouri et al. [32] suggested Hybrid COASaDE as a mixed optimization technique, which combined the exploration capabilities of the Crayfish Optimization Algorithm and adaptive exploration of self-adaptive DE. This combination of global and local designs guarantees a symmetric tradeoff between overall search and local refinement by dynamic parameter control. A neural network-assisted hybrid version of the Hiking Optimization Algorithm (HOA), which incorporated chaotic dynamics and self-designed AI optimizations to enhance design optimization in engineering, was presented by Ozcan et al. [33]. To overcome these limitations, Xia et al. [34] proposed a Fractional Order Dung Beetle Optimizer with Reduction Factor (FORDBO) that enhances the standard DBO algorithm through good node set initialization, a reduction factor for exploration–exploitation balance, fractional order calculus for dynamic boundary adjustment, and a repetitive renewal mechanism, demonstrating superior performance against 23 competing algorithms.

The Parrot Optimization Algorithm (PO) [35] is a recent MA that is based on the observation of behavior in the Pyrrhura Molinae parrot species. This algorithm is based on computational principles derived from the behaviors of the parrot, such as its foraging, resting, social communication, threat avoidance, and activity inhibition. The PO methodology utilizes a multi-stage approach where it copies parrots’ nesting habitats using the spatial surroundings, then models their social behaviors and adaptive plasticity to environmental stimuli. Parrots show behavioral characteristics of social cooperators and environmental generalists and inhabit an intricate ecological niche. PO exploits these behavioral properties by further exploiting environmental fluctuations and social interactions in its computational framework to solve optimization problems with high complexity and varied constraints. The algorithm demonstrates efficacy in problem-solving as it dynamically adjusts to the problem landscape using a simulation of parrot behavioral strategies.

Although PO has an acknowledged application in resolving engineering optimization challenges [36,37,38,39], it faces limitations rendering the need to establish an improved variant in this study. First, original PO works satisfactorily in some functions but shows weaknesses such as poor accuracy and conciseness when faced with non-convex and high-dimensional optimization problems. Moreover, it also has difficulties striking optimal tradeoffs between exploration and exploitation stages that lead to suboptimal solutions in hard optimization problems that feature complex search landscapes and have multiple constraints. Secondly, with ongoing optimization challenges in different fields with the features of high dimensionality, large search spaces, and nonlinearity, current MAs often face problems of stability and convergence. This requires consistent research activities to develop new and work on the advancement of current MAs to solve computational challenges. Thirdly, the NFL theorem suggests that no MA can always be preferential throughout all optimization problems. As a consequence, there is a necessity for new and dynamic solutions to optimize the performance of algorithms and overcome existing constraints. Finally, recent algorithmic advances in algorithmic design and optimization algorithms point out that it is imperative to explore optimization algorithms and refine them to better serve the demands of practical computational optimization problems. Thus, the proposed improvements to PO described in this paper are based on these motivations.

Based on the above justification and to directly address the identified research gaps, the paper integrates three superior approaches, including Mean Differential Variation (MDV), Dimension Learning-Based Hunting (DLH), and Enhanced Adaptive Mutualism (EAM), into PO and suggests modified Adaptive Differentiated Parrot Optimization (ADPO), with improved capacities of performance. To address the diversity loss and premature convergence gap, the MDV strategy is used to rectify the loss of the generation of population diversity into the creation of a dual-phase mutation mechanism that fosters wide exploration in initial iterations and heavy exploitation near optimal solutions in later iterations. This strategy provides optimization of many different solutions as well as a high convergence rate. Moreover, to tackle the insufficient dimension-wise learning gap, the DLH strategy is presented in an improved manner that avoids premature convergence, as each solution is allowed to adaptively learn from its dimension-wise neighbors. This strategy contributes to the creation of diverse neighborhoods because each solution has a local neighborhood with spatially close neighbors, thus facilitating coordinated but individually directed search. Also, to overcome the rigid cooperation mechanism gap, we implement an EAM strategy. Thus, flexible elite or random and fitness-based influence are used in this strategy to keep a good balance between intensification and diversification during the optimization process.

These three strategies, when combined with PO, effectively tackle the inherent problems of unbalanced exploration–exploitation, few correct solutions, and low convergence speed. ADPO has been stringently compared to a large number of classical and state-of-the-art algorithms across several benchmark suites, including CEC2017 and CEC2022 test functions, and rigorous statistical analysis using Friedman rank tests [40], Wilcoxon signed-rank tests [41], and broad convergence assessment. The outcomes present an excellent improvement in performance compared to that of original PO, with ADPO being notably competitive against other advanced algorithms. The applicability of the enhanced ADPO technique is also depicted in this paper by integrating it with LSTM networks to predict wind power as a real-world application. The ADPO-LSTM hybrid architecture helps solve the most important problem of hyperparameter search in renewable energy forecasting systems and shows better performance in measuring wind farm stations. In these applications, ADPO maintained outstanding stability and accuracy. Thus, the primary contributions of this paper are summarized as follows:

- An excellent improvement of PO, which is known as ADPO, is presented. This variant integrates MDV, DLH, and EAM to address high-dimensional complex optimization problems in a highly efficient manner.
- The DLH strategy enhances the population diversity and boosts the exploration abilities of PO without premature convergence.
- An EAM mechanism is designed to overcome the fixed cooperation with fitness-directed interaction, which enhances balance in intensification and diversification during the optimization process.
- The MDV strategy is used to enhance both the exploration and exploitation ability in diversity loss because of mutation by the dual-phase strategy, which preserves convergence power.
- ADPO is specifically designed to enhance convergence speed and solution accuracy while effectively avoiding local optima through comprehensive diversity preservation mechanisms.
- Thorough numerical tests against the other main intelligent algorithms and robust optimizers on the CEC2017 and CEC2022 test suites have shown that ADPO comports exceptionally well in solving the various optimization problems in multiple dimensions.
- ADPO could be successfully applied to the LSTM neural networks in the wind power forecasting, representing the state-of-the-art results and indicating the feasible applicability in the renewable energy systems.

This paper is structured as follows: Section 2 describes the basic principles and mathematical formulation of the Parrot Optimization Algorithm and detailed algorithms. In Section 3, improved ADPO is introduced, where the three improvement strategies are elaborated in detail with their mathematical expressions and implementation processes. The results of extensive numerical experiments on the CEC2017 and CEC2022 benchmark suites in various dimensions, where a detailed statistical analysis and an evaluation of the performances are reported, are presented in Section 4. Section 5 introduces the ADPO-LSTM hybrid model of a wind power forecasting model with experimental evaluation of the performance on actual wind electricity farm data. Section 6 concludes the research findings and provides desirable future research directions in the development of new types of renewable energy applications and optimization algorithms.

## 2. Parrot Optimization Algorithm (PO)

The Parrot Optimization Algorithm (PO) is a nature-based computing technique which is based on the observation of the behavioral patterns of a sociable parrot species, Pyrrhura Molinae [35]. The main mechanics of this algorithm are based on replicating five different behavioral aspects common in these birds: food search, periods of rest, communication among solutions, avoiding new threat sources, and ultimate cessation of activity. All these automatic behaviors are mapped into operational steps in the algorithm to dynamically balance exploration of new areas and exploitation of promising regions.

### 2.1. Phase 1: Population Initialization

When the algorithm starts, a population of agents is distributed at random in the search domain. The initial position of each solution, which represents a parrot in the decision space, is determined via a uniformly distributed random generator. The equation used for this initialization is as follows:

(1) $$X_{i}(0)=lb+rand(0,1)\cdot (ub-lb)$$

Here, $X_{i}(0)$ is the starting position of the i-th parrot. The terms lb and ub are used to refer to the previously defined low and high limits of the optimization variables, respectively. The term rand(0, 1) generates a random number that falls between 0 and 1, and therefore, the generated solution will not go beyond the range specified. Such random initialization creates adequate diversity in the population and lays the foundation for wide initial exploration of the problem space.

### 2.2. Phase 2: Foraging

During this critical phase, each solution actively explores the search space in pursuit of improved solutions, akin to a parrot seeking food. The solution’s movement is directed by the best solution discovered so far $X_{\mathrm{best}}$ and the average location of all solutions $X_{\mathrm{mean}\;}(t)$. To encourage exploration and prevent stagnation, a Lévy distribution is used to add stochastic perturbations. The position of each solution is adjusted by

(2) $$X_{i}(t+1)=\left(X_{i}(t)-X_{\mathrm{best}}\right)\cdot Levy(dim)+rand(0,1)\cdot {\left(1-t/T_{max}\right)}^{\left(2t/T_{max}\right)}\cdot X_{\mathrm{mean}}(t)$$

Here, $X_{i}(t)$ is the position of the i-th solution at iteration t, and $T_{\max}$ defines the maximum number of iterations. The component ($X_{i}(t)-X_{\mathrm{best}}$) quantifies how far the solution is from the best-known solution, driving it toward better regions. The term Levy(dim) facilitates both small and large steps, enabling the algorithm to escape local optima. The global behavioral tendency is incorporated through $X_{\mathrm{mean}\;}(t)$ which is computed as

(3) $$X_{\mathrm{mean}\;}(t)=\frac{1}{N}\sum_{k=1}^{N}X_{k}(t)$$

where N is the number of parrots (solutions). This averaging ensures some cohesion in the flock’s movement while preserving diversity.

### 2.3. Phase 3: Resting

Parrots take breaks at times, particularly when they are around friends. The relaxation step replicates this action by moderately controlling agent motion without halting the optimization process. Instead of moving toward distant regions, solutions are found close to the optimal solution, and they engage in small position changes. The formula for updating is as follows

(4) $$X_{i}(t+1)=X_{i}(t)+X_{\mathrm{best}}\cdot Levy(dim)+rand(0,1)\cdot ones(1,dim)$$

In this case, ones(1,dim) takes a small vector of ones of all dimensions, together with a random scalar which adds some weak noise to all coordinates when multiplied. This phase facilitates exploitation without hindering the convergence process of the population.

### 2.4. Phase 4: Communication

Parrots are social creatures. During the algorithmic process, solutions exchange and synthesize information with other members of the population. Communication behavior is applied probabilistically as indicated by a random variable Q, which specifies the probability of one agent aligning with the group or acting independently. The new location can be calculated as follows:

(5) $$X_{i}(t+1)=\left\{\begin{array}{ll} 0.2\cdot rand(0,\;1)\cdot \left(1-t/T_{max}\right)\cdot \left(X_{i}(t)-X_{mean}(t)\right), & Q\leq 0.5\\ 0.2\cdot rand(0,\;1)\cdot exp\left(-t/\left(rand(0,\;1)\cdot T_{max}\right)\right), & Q>0.5 \end{array}\right.$$

When Q≤0.5, the parrot’s movement is influenced by the mean of the population, modeling collaborative behavior. If Q>0.5, the solution moves away with exponential decay, signifying independence after group interaction. These dynamics foster both cohesion and divergence, which is critical for sustaining balance between exploration and convergence.

### 2.5. Phase 5: Strange Aversion

Parrots avoid strange animals and prefer staying close to familiar companions. Solutions are driven toward favorable areas and pushed away in less promising or fraudulent areas, using this as a natural defense mechanism in the algorithm. The update of the position is carried out as follows:

(6) $$X_{i}(t+1)=X_{i}(t)+rand(0,1)\cdot cos\left(0.5\pi -\frac{t}{T_{max}}\right)\cdot \left(X_{\mathrm{best}}-X_{i}\left(t\right)\right)-cos(rand(0,1)\cdot \pi )\cdot {\left(\frac{t}{T_{max}}\right)}^{\frac{2}{T_{max}\;}}\cdot \left(X_{i}(t)-X_{\mathrm{best}}\right)$$

This equation comes up with a dual mechanism. The initial cosine term favors motion towards the optimal solution, with time-dependent modulation. The second cosine term produces repelling motion, subjected to randomness that prevents stagnation. This is a critical phase in reifying exploitation of the most familiar solution without impairing population diversity and strength.

### 2.6. Phase 6: Termination Condition

As soon as the calculated iteration $T_{max}$ is achieved (a maximum number of iterations), the algorithm stops. This stage makes sure that optimization is performed under the computational terms. At this moment, the most optimal solution found by the swarm should be adequately set.

## 3. Proposed Adaptive Differentiated PO (ADPO)

Although original PO can be attractive when it comes to balancing exploration and exploitation in nature-inspired optimization, the algorithm has a number of fundamental weaknesses. The population diversity decreases at a very fast pace as the search proceeds, putting the search at risk of premature convergence and hindering the escape from local optima. The inherent rigidity of mutualistic cooperation also limits adaptability, and dimension-level learning is ineffective in high-dimensional problems. To combat such issues, advanced ADPO incorporates three novel methods, described as follows:

- **Mean Differential Variation (MDV):** This overcomes the early loss of diversity by introducing a two-phase mutation mechanism that promotes wide exploration in early iterations and intensifies searching near the best solutions in later stages.
- **Dimension Learning-Based Hunting (DLH):** This prevents premature convergence by enabling each solution to adaptively learn from dimension-wise neighbors, promoting diversity and enabling coordinated yet independent directional search.
- **Enhanced Adaptive Mutualism (EAM):** This integrates the rigid mutualism of PO with an adaptive cooperation model that uses fitness-based influence and flexible references to maintain balance between intensification and diversification.

These strategies collectively enhance ADPO’s global search capability, resilience against local optima, and convergence reliability, particularly in complex optimization landscapes.

### 3.1. Mean Differential Variation (MDV)

When PO is being optimized, there is a tendency for the population to converge on elite solutions. This increases convergence speed significantly, but usually leads to a quick decrease in diversity. Such diversity loss prematurely homogenizes the population, causing stagnation and local optima entrapment.

To overcome this drawback, ADPO integrates the mechanism of MDV. This strategy proposes two adaptive update formulae, which are used at various stages of the optimization. In both variants, two agents, $X_{r1}$ and $X_{r2}$, are randomly selected from the population. Two auxiliary vectors are calculated as follows:

(7) $$X_{c1}=\frac{X_{r1}+X_{r2}}{2},X_{c2}=\frac{X_{r1}+X_{best}}{2}$$

In this formulation, $X_{\mathrm{best}}$ denotes the globally best solution discovered so far. The vectors $X_{c1}$ and $X_{c2}$ serve as hybrid guides for movement, combining information from both random and elite individuals. During the first two-thirds of the total iteration budget, i.e., when $t<\frac{2}{3}T_{max}$, the update equation promotes exploration and is defined as follows:

(8) $$X_{i}=X_{c1}+F\cdot \left(X_{c1}-X_{i}\right)+F\cdot \left(X_{c2}-X_{i}\right)$$

Here, $X_{i}$ is the i-th individual and F is a constant scaling factor set to 0.25. This mechanism directs solutions toward unexplored regions derived from intermediate positions. Once the algorithm enters its final-third phase of iterations, the emphasis switches to local refinement. The position update for $X_{i}$ becomes

(9) $$X_{i}=X_{best}+F\cdot \left(X_{c1}-X_{i}\right)+F\cdot \left(X_{c2}-X_{i}\right)$$

In this later stage, the scaling factor F becomes dynamic and is calculated as F=(1−2⋅rand(1))⋅0.5. This randomized scaling helps adjust movement intensity near $X_{\mathrm{best}}$, enabling fine-tuning without eliminating randomness. Thus, the full mutation rule across both phases is summarized as

(10) $$\;\mathrm{If}\;t<\frac{2}{3}T_{max}:F=0.25,X_{i}=X_{c1}+F\left(X_{c1}-X_{i}\right)+F\left(X_{c2}-X_{i}\right)\;\mathrm{Else}:\;F=(1-2\cdot rand(1))\cdot 0.5,X_{i}=X_{\mathrm{best}}+F\left(X_{c1}-X_{i}\right)+F\left(X_{c2}-X_{i}\right)$$

The MDV strategy can optimize the search process because it supports wider motions in the initial stage and precise exploitation in the final one. This two-phase behavior serves to maintain diversity in the beginning and concentrate efforts in the vicinity of prospects at a later stage. The consequence of this in ADPO is a better global search and solution enhancement balance, leading to accelerated convergence and better solutions.

### 3.2. Dimension Learning-Based Hunting Search Strategy (DLH)

In PO, the characteristic of the solutions converging towards the best-known solution quickly can be problematic because of high-dimensional search landscapes. The fewer solutions are less diverse as iterations continue. Such a reduction in the population search space limits the potential of the algorithm to search other areas, leading to a greater possibility of getting trapped in local optima. This behavior gets accentuated more when resolving complex multimodal problems.

To counteract this loss of diversity and keep a strong exploratory capacity throughout the search, the DLH strategy is added to the proposed ADPO. Moreover, DLH promotes diversity in the population and the sharing of information by ensuring that every solution has a customized neighborhood along the lines of spatial closeness. Such neighborhoods enable solutions to learn adaptively under a choice set of peers, thereby facilitating collaborative, though focused, search practices. First, the algorithm starts by calculating a new position $X_{i}(t+1)$ for the i-th parrot, with the help of already-existing PO’s original operators. Then the Euclidean distance between the current location and the new position is calculated as follows:

(11) $$R_{i}(t)=\left\|X_{i}(t)-X_{i}(t+1)\right\|$$

This distance value $R_{i}(t)$ helps define a local neighborhood around $X_{i}(t)$, denoted as $N_{i}(t)$, which includes all individuals $X_{j}(t)$ whose distance from $X_{i}(t)$ is less than or equal to $R_{i}(t)$, which can be modeled as follows:

(12) $$N_{i}(t)=\left\{X_{j}(t)|\left\|X_{i}(t)-X_{j}(t)\right\|\leq R_{i}(t),X_{j}(t)\in \;\mathrm{Population}\;\right\}$$

Once the neighborhood $N_{i}(t)$ is established, a dimension-wise learning mechanism is employed. For each dimension d, a new candidate solution $X_{i,d}(t+1)$ is generated using one randomly chosen neighbor $X_{n,d}(t)\in N_{i}(t)$ and a random individual $X_{r,d}(t)\in$ population. The updated dimension is computed as follows:

(13) $$X_{i,d}(t+1)=X_{i,d}(t)+rand\cdot \left(X_{n,d}(t)-X_{r,d}(t)\right)$$

This equation promotes both collaboration (via neighbor $X_{n}$) and stochastic variation (via $X_{r}$), encouraging adaptive updates tailored to the current search state. After producing both the original candidate $X_{i}(t+1)$ and the dimension-learned candidate $X_{id}(t+1)$, their fitness values are compared, where the best one is chosen for the next iteration as follows:

(14) $$X_{i}(t+1)=\left\{\begin{array}{ll} X_{i}(t+1), & \;\mathrm{if}\;f\left(X_{i}(t+1)\right)<f\left(X_{id}(t+1)\right)\\ X_{id}(t+1), & \;\mathrm{otherwise}\; \end{array}\right.$$

This selection process ensures that only the more promising of the two solutions is retained, further refining the solution’s path.

It is worth noting that the combination of allowing each solution to learn individually and locally and having directionally dissimilar solutions qualifies DLH as a mechanism that can prevent the premature closure of the search space. Furthermore, this mechanism boosts the algorithmic potential of exploring various dimensions and, at the same time, makes good use of the information on the surrounding solutions. Consequently, ADPO ensures wider diversity between its iterations, and hence, results in better resilience to local optima and more robust convergence towards the global optimum overall.

### 3.3. Enhanced Adaptive Mutualism (EAM) Strategy

ADPO employs the EAM strategy in order to enhance the adaptive capacity and cooperation dynamics among solutions. This enhanced mechanism allows more biologically plausible interactions in which the effect of partners depends on their fitness and in which the partners in cooperation are selected flexibly. The cooperative interaction begins by selecting two individuals: $X_{i}(t)$, the current solution, and $X_{z}(t)$, a randomly chosen partner from the population. A mutual vector is computed to represent their midpoint, as follows:

(15) $$MV=\frac{X_{i}(t)+X_{z}(t)}{2}$$

Next, a decision is made regarding the cooperation target. If a uniformly sampled variable q∈[0,1] is less than 0.5, the guiding reference becomes $X_{\alpha }$, which is the mean of the top three best-performing individuals, calculated as follows:

(16) $$X_{\alpha }=\frac{X_{{\mathrm{best}\;}_{1}}+X_{{\mathrm{best}\;}_{2}}+X_{{\mathrm{best}\;}_{3}}}{3}$$

In this case, the position of both participants is updated as follows:

(17) $$X_{i}(t+1)=X_{i}(t)+rndm\cdot \left(X_{\alpha }-MV\cdot BF_{1}\right)$$

(18) $$X_{z}(t+1)=X_{z}(t)+rndm\cdot \left(X_{\alpha }-MV\cdot BF_{2}\right)$$

Alternatively, if q≥0.5, the cooperation target is a randomly selected individual $X_{r}$, and the update becomes

(19) $$X_{i}(t+1)=X_{i}(t)+rndm\cdot \left(X_{r}-MV\cdot BF_{1}\right)$$

(20) $$X_{z}(t+1)=X_{z}(t)+rndm\cdot \left(X_{r}-MV\cdot BF_{2}\right)$$

In both equations, rndm∈[0,1] is a uniform random value that controls the step size of movement, introducing stochasticity into the update. The benefit factors $BF_{1}$ and $BF_{2}$ are not fixed but computed based on the fitness of each participant relative to the global best, as follows:

(21) $$BF_{1}=\frac{f\left(X_{i}\right)}{f\left(X_{\mathrm{best}}\right)}\;\mathrm{if}\;f\left(X_{\mathrm{best}}\right)\neq 0$$

(22) $$BF_{2}=\frac{f\left(X_{z}\right)}{f\left(X_{\mathrm{best}}\right)}\;\mathrm{if}\;f\left(X_{\mathrm{best}}\right)\neq 0$$

These adaptive benefit values allow solutions with higher fitness to exert stronger mutual influence, leading to more meaningful and responsive cooperation patterns.

**Figure 1:**
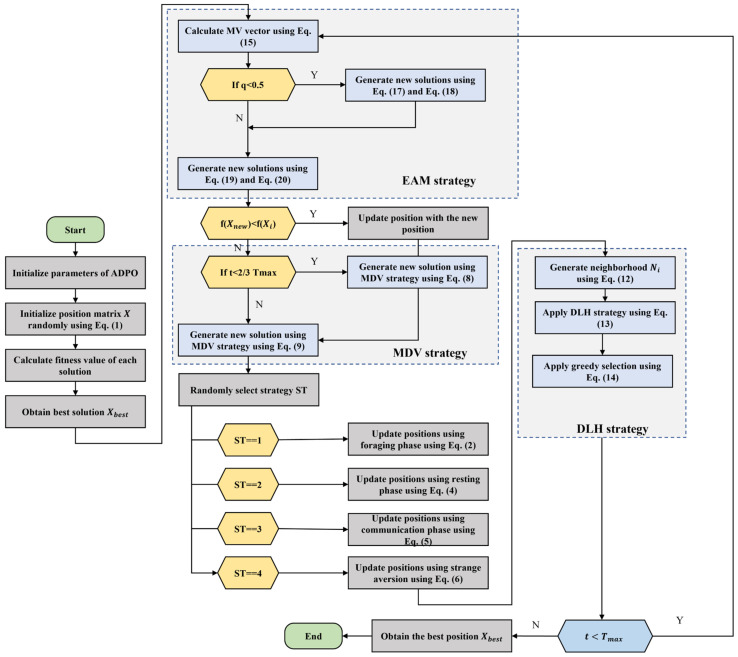
Proposed ADPO.

This mechanism enhances ADPO performance considerably by improving the balance mechanism between intensification and diversification. Elite solutions tend to be followed, and the population therefore exploits high-potential regions. Thus, the algorithm becomes exploratory when random references are selected. Moreover, the benefit scaling based on fitness renders the strength of interaction context-sensitive and free of inefficient or redundant movements. Therefore, the EAM strategy makes ADPO much more able to avoid getting trapped in misleading regions, more likely to converge, and more able to explore advantageous regions of the search landscape effectively. The main steps of the proposed ADPO are presented in Figure 1 and Algorithm 1. The overall ADPO framework shows how the three proposed enhancement strategies are seamlessly integrated into the optimization process. The framework demonstrates the sequential application of EAM for population updating, followed by MDV for diversity preservation, then the original PO operators, and finally DLH for dimension-wise learning.
**Algorithm 1: ADPO****Input:**    **Maximum number of iterations** 
$T_{max}$
**,**
    **Population size** 
N
**,**
    **Number of dimensions** 
d
**,**
    **Upper bound** 
ub and lower bound lb
**.**
**Output:**
    **Optimal solution** 
$X_{best},\;fitness\;value\;of\;optimal\;solution\;f_{best}$
1. Initialize the initial population i←1,2,….,N Equation (1)2. Evaluate the fitness value of each solution $f(X_{i})$3. Obtain the best solution $X_{best}$ and its fitness value $f_{best}$4. **while** $t\leq T_{max}$ **do**5.    Obtain the best solution $X_{best}$ and its fitness value $f_{best}$6.    **for** i=1 to N **do**7.     Generate new solution $X_{new}$ using EAM strategy using Equations (17)–(20)8.     **if** $f\left(X_{new}\right)<f(X_{i})$ **then**9.      
$f\left(X_{i}\right)=f(X_{new})$
10.     
**end if**
11.    **end for**
12.    **for** i=1 to N
**do**13.     Generate new solution $X_{new}$ using MDV strategy using Equations (8) and (9)14.    **end for**
15.    **for** i=1 to N
**do**16.     ST = $randi(\left[1\;4\right])$17.     
**if** 
ST==1 **then**
18.      Update the position of solution using Equation (2)19.     
**Elseif** 
ST==2 **then**
20.      Update the position of solution using Equation (4)21.     
**Elseif** 
ST==3 **then**
22.      Update the position of solution using Equation (5)23.     
**Elseif** 
ST==4 **then**
24.      Update the position of solution using Equation (6)25.      **end if**
26.    
**end for**
27.    **for** i=1:N **do**28.     Apply DLH strategy for each solution using Equations (11)–(13)29.     Apply greedy selection30.    **end for**
31.    Check if the solution within the defined boundary and calculate fitness values.32.    Update the best solution found $X_{best}$33. **end while**34. **Return** $X_{best}$ and its fitness value $f_{best}$;

## 4. Analysis of Global Optimization Performance

The section includes an extensive experimental analysis of ADPO with all the details of the experimental configuration and a comparison with the results of competing algorithms in terms of their performance. The test methodology compares the behavior of the algorithms on the benchmark suites CEC2017 [42] and CEC2022 [43], which include representative test cases that emulate real-world optimization problems. This section confirms the effectiveness of ADPO with the help of pattern convergence and displaying statistical distributions. Also, extensive comparisons with state-of-the-art MAs demonstrate that ADPO effectively handles high-dimensional and complex optimization problems.

### 4.1. Experimental Configuration and Settings

To develop ADPO’s performance evaluation, we leveraged two well-known benchmark suites—the CEC2017 and CEC2022 test functions—commonly used to assess the effectiveness of optimization algorithms. The CEC2017 suite includes 29 functions that are divided into unimodal functions (F1, F3), multimodal functions (F4–F10), and hybrid functions (F11–F20), as well as composition functions (F21–F30). The functions in the suite used in CEC2022 are 12 of the most complex optimization problems. In CEC2017 and CEC2022, all benchmark functions are assembled on [−100, 100]. These measurement standards provide an in-depth test of the capabilities of exploration globally and exploitation locally by utilizing strict test procedures. The dimensionalities are 50 in the CEC2017 functions and 20 in the CEC2022 functions. The size of both benchmark suites remained at a fixed value of 30 individuals, and each algorithm ran 30 independent trials to offer statistical reliability. The computational experiments were conducted on a system containing an Intel Core i7-10750H processor outputting 2.60 GHz, supported with 32 GB of RAM, on the Windows 10 (64-bit) operating system. The MATLAB R2024b environment was applied to implement and execute the algorithms.

The optimization algorithms which were chosen to undergo the comparative analysis are powerful MAs that have shown outstanding results in various optimization fields. The comparison framework involves the following algorithms: (1) Classical MAs, which have a history of validation, such as the Arithmetic Optimization Algorithm (AOA) [44], Harris Hawk Optimization (HHO) [45], Whale Optimization Algorithm (WOA) [46], and original Parrot Optimization (PO). These foundational algorithms have proven to be robust when dealing with a variety of optimization problems. (2) Recent research works in metaheuristic optimization, such as the Spider Wasp Optimizer (SWO) [47], Golden Jackal Optimization (GJO) [48], Coati Optimization (COA) [49], IVY algorithm [50], Hiking Optimization Algorithm (HOA) [27], and Kepler Optimization Algorithm (KOA) [51]. These new algorithms present new approaches and techniques for solving complex problems. (3) High-performance optimization algorithms, including competition-winning and state-of-the-art techniques, including eCOA [52], CMAES [53], GWO_CS [54], RDWOA [55], CSOAOA [56], DAOA [57], ISSA [58], jDE [59], and IPSO_IGSA [60]. These advanced algorithms are state-of-the-art optimization technologies that possess excellent performance attributes. Detailed settings for each algorithm are listed in Table 1.

Selection of comparative algorithms followed strict criteria based on relevance and proven efficiency in terms of optimization studies and proven efficiency in solving various problems in optimization. The classical algorithms offer basic metrics that represent established methods of approaches developed long ago, whereas the recent techniques demonstrate newer emerging strategies. State-of-the-art techniques are represented by advanced algorithms along with solutions that have proved to be optimal in terms of the competition, which guarantees that all the competition will be evaluated as thoroughly as possible regarding the most advanced optimization technologies. This structured selection ensures exhaustive evaluation of ADPO across classical, modern, and advanced optimization paradigms.

### 4.2. Metrics for Evaluating Optimization Performance

In order to impartially assess the quality of work of ADPO among these different rivals, we used a number of statistical and descriptive measures. These are the average quality of solutions (AVG), standard deviation (SD), Friedman ranking (FR), and the Wilcoxon rank-sum significant test. All these metrics have their own role in the profiling of the consistency, reliability, and superiority of the optimizer.

- **Mean Fitness (AVG):** The measure of the quality of solutions typically attained is the average fitness score over different independent runs. This measurement is useful in the evaluation of the correctness and general performance of an algorithm in repetitive usage within the same setup. It is calculated as follows:

(23) $$AVG=\frac{1}{N}\sum_{i=1}^{N}f_{i}$$

where N is the number of runs and $f_{i}$ is the fitness value obtained in the $i^{th}$ trial.

- **Standard Deviation (SD):** SD quantifies the extent of dispersion of the fitness values around the mean, providing information about the consistency and stability of the results produced by the algorithm. Smaller variations show that the optimizer provides consistent results when repeated many times. It can be calculated as follows:

(24) $$SD=\sqrt{\frac{1}{N}\sum_{i=1}^{N}\;{\left(f_{i}-AVG\right)}^{2}}$$

- **Friedman Ranking (FR)** [40]: This nonparametric statistical test ranks algorithms based on their relative performances across multiple problem instances. A lower average rank suggests superior performance. The final ranking is derived from averaging ranks over all tested functions. The Friedman test statistic is then evaluated using a chi-squared distribution to determine consistency in relative performance across functions.
- **Wilcoxon Rank-Sum Test** [41]: To establish whether performance differences between ADPO and any competing algorithm are statistically meaningful, the Wilcoxon rank-sum test is utilized. A p-value below 0.05 denotes a significant difference. If ADPO achieves better results, it is marked with R+; if no clear difference exists, it is annotated with R=; and if ADPO underperforms, it is labeled with R−.

The combination of these metrics provides an extensive basis to compare ADPO to other algorithms and make sure that all findings regarding the optimization performance of this algorithm are statistically aligned and indicative of its practical potential.

### 4.3. Ablation Study

The primary objective of the ablation study is to systematically evaluate the individual contributions of each proposed enhancement strategy within ADPO and quantify their specific impact on optimization performance [61]. This analysis aims to understand how each mechanism individually contributes to the overall superiority of ADPO compared to the original PO. To conduct this comprehensive analysis, we designed five distinct algorithmic variants: (1) ADPO: the complete proposed algorithm incorporating all three enhancement strategies; (2) ADPO-MDV: a variant incorporating only the MDV strategy while maintaining the original PO framework for other operations; (3) ADPO-DLH: a variant implementing only the DLH mechanism with standard PO operations; (4) ADPO-EAM: a variant utilizing only the EAM strategy combined with original PO components; (5) PO: the baseline original PO for comparison. Each variant was evaluated using the CEC2022 benchmark suite comprising 12 functions with diverse characteristics: F1 (unimodal), F2–F4 (multimodal), F5–F8 (hybrid), and F9–F12 (composite functions). Table 2 presents the comprehensive ablation study results, revealing significant insights into the individual and collective effectiveness of each proposed enhancement strategy across all benchmark functions.

- **Enhanced Adaptive Mutualism (EAM) strategy analysis:** The ablation study reveals that ADPO-EAM emerges as the most impactful individual enhancement, achieving an average rank of 1.75 and securing first place on four functions (F1, F9, F11, F12). This strategy demonstrates exceptional performance on unimodal function F1, with the best average fitness (300.839) and remarkably low standard deviation (0.390), indicating superior exploitation capability and convergence stability. On composition functions F9, F11, and F12, ADPO-EAM consistently outperforms other individual strategies, showcasing its effectiveness in handling complex multimodal landscapes through adaptive fitness-guided cooperation. The substantial improvement over the original PO validates the critical importance of flexible mutualistic interactions in optimization performance.
- **Mean Differential Variation (MDV) strategy analysis:** ADPO-MDV demonstrates moderate but consistent improvements, with an average rank of 3.25, representing significant enhancement over baseline PO. This strategy shows particular strength in maintaining solution quality across diverse function types, with notable performance on multimodal functions F2–F4, where it consistently ranks third. The dual-phase mutation mechanism effectively balances exploration and exploitation, as evidenced by its reasonable standard deviation values and stable performance across all function categories. However, the strategy shows limitations on more complex hybrid and composition functions, suggesting that while MDV provides valuable diversity preservation, it requires synergistic combination with other mechanisms for optimal performance in challenging optimization landscapes.
- **Dimension Learning-Based Hunting (DLH) strategy analysis:** ADPO-DLH achieves an average rank of 4.17, showing the most limited individual impact among the three proposed strategies. Interestingly, this strategy demonstrates selective effectiveness, performing competitively on hybrid function F5 (rank 2) while showing poor performance on other function types, particularly unimodal F1, where it ranks fourth. The high standard deviation values observed in several functions (notably F1 and F6) indicate instability in convergence behavior when used in isolation. This pattern suggests that DLH’s dimension-wise learning mechanism requires the stabilizing influence of other strategies to achieve consistent performance, validating its role as a complementary rather than standalone enhancement.

The complete ADPO framework achieves the best overall performance, with an average rank of 1.33 and a Friedman rank of 1.55, demonstrating superior synergistic effects when all three strategies operate collectively. ADPO secures first place on eight out of twelve functions (F2, F3, F4, F5, F6, F7, F8, F10), showcasing consistent excellence across all function categories. Most remarkably, ADPO achieves the lowest standard deviation values on most functions, indicating exceptional stability and reliability compared to individual strategy implementations.

The Friedman rank analysis confirms statistically significant differences between all algorithmic variants, validating the reliability of the observed performance differences. The substantial rank gap between the complete ADPO framework (1.55) and individual strategies (1.87 to 4.00) provides strong statistical evidence for the necessity of integrated enhancement mechanisms. Furthermore, the consistent superiority of all enhanced variants over original PO (Friedman rank 4.34) confirms that each proposed strategy contributes meaningful improvements to the baseline optimization capability. This comprehensive ablation study conclusively demonstrates that while each individual enhancement strategy provides valuable improvements over original PO, the complete ADPO framework is the best-performing.

### 4.4. Results Discussion Using CEC2022

The main goal of this section is to test and compare the performance of the proposed ADPO with a large number of well-known optimization algorithms. The comparison was performed with the CEC2022 benchmark suite to evaluate the different capabilities of the algorithms. The obtained results are presented in Table 3.

The unimodal function F1 tests the algorithms’ exploitation capability and convergence speed toward a single global optimum. As shown in Table 3, ADPO demonstrates exceptional performance, with an average fitness value of 301.302 and a remarkably low standard deviation of 0.981, securing the first rank. This outstanding performance indicates ADPO’s superior ability to intensify search around promising regions while maintaining consistent convergence behavior. Original PO ranks third with a significantly higher average fitness (1.60 × 10^4^), highlighting the substantial improvements achieved through the proposed enhancements. The large performance gap between ADPO and other competitors, particularly KOA (1.11 × 10^5^), which ranks last, demonstrates the effectiveness of the MDV strategy in achieving precise exploitation during the later stages of optimization.

On the other hand, the multimodal functions F2–F4 test the algorithms’ exploration ability and capacity to get out of local optima in order to find global solutions. For F2, it can be seen that ADPO ranks second, with an average fitness of 466.259, after IVY (464.089), which came first. The close performance level of these best performers implies that the DLH strategy of ADPO is successful in sustaining population diversity. In the case of F3, original PO achieved the rank of fifth (655.570), and ADPO ranked second (621.667) following IVY, which demonstrates increased exploration. On F4, ADPO ranks second (878.170) once more, with IVY in the first position, indicating reliable performance in various multimodal terrains. The high output, in contrast to the classical methods such as WOA (932.687, place 7) and more advanced methods such as KOA (1063.219, place 11), proves the efficiency of the proposed enhancement patterns.

Furthermore, hybrid functions are functions mixing properties of several types of functions and often generate sophisticated optimization landscapes that may hamper exploration and exploitation capabilities. On F5, Table 3 indicates that ADPO is in position two (2013.369), after GJO (1900.620), demonstrating a competitive nature in dealing with hybrid complexities. The algorithm is much better than the original PO (2518.767, rank 4) and classical approaches such as WOA (3919.915, rank 10). In the case of F6, ADPO has the highest performance, with a fitness value of 4028.385 and the first rank, showing an excellent performance in challenging hybrid landscapes. This excellent result, contrasting with the abject failures of some of the competitors, such as KOA (3.43 × 10^9^), testifies to the strength of the EAM strategy approach. ADPO holds first and second places on F7 and F8 with fitness values of 2103.721 and 2243.629, respectively, outperforming the majority of competitors and demonstrating better suitability to handling the complexities of hybrid functions.

Finally, the composition functions are the most difficult optimization situations, where many functions with various properties, global optima, and local topologies come together. Table 3 shows that ADPO has impressive performance on all composition functions, ranking first on F9, F10, F11, and F12, respectively, with fitness values of 2481.000, 2539.901, 2935.671, and 2991.295, respectively. Specifically, ADPO produced an outstanding score on F9 despite having the lowest average fitness; it was nearly perfectly consistent (standard deviation of 0.282). For F10, the large performance difference between ADPO and the second-ranking PO (2778.659) depicts the better capability of the algorithm when addressing a complex landscape of compositions. The stability of its first ranking across all the composition functions proves that all three suggested augmentation measures work in synergy to address the hardest optimization cases.

Thorough analysis shows that ADPO achieved a median rank of 1.42 across the total number of test functions, and it is in the top place overall, as indicated in Table 3. This outstanding performance is a big step up from the original PO (average rank 3.83, final rank 4) and clearly superior to every other algorithm. The IVY algorithm obtains second place with an average rank equaling 3.08, whereas classical algorithms such as WOA (average rank 6.33) and recent ones such as HOA (average rank 7.58) perform significantly worse. The stability of ADPO in ranking across a wide variety of types of functions suggests the resilience and versatility of the algorithm in different optimization landscapes.

The conducted statistical measurement via standard deviations shows the remarkable consistency of ADPO, especially in the F1 and F9 functions, where the algorithm records very low standard deviations of 0.981 and 0.282, respectively. The combination of such consistency means that ADPO is not only more likely to discover high-quality solutions, but also to do so in a consistent fashion across independent runs. The suggested optimization approach, including the MDV, DLH, and EAM strategies, combines to correct the drawbacks of the original PO and preserve their own advantages, leading to a powerful and supportive optimization algorithm that can be used in a variety of real-life applications.

**Figure 2:**
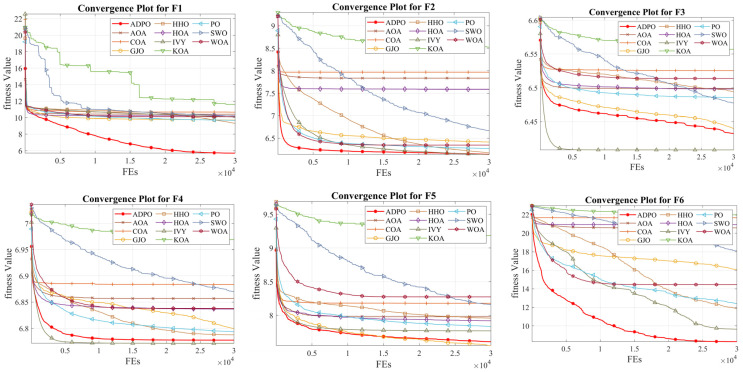

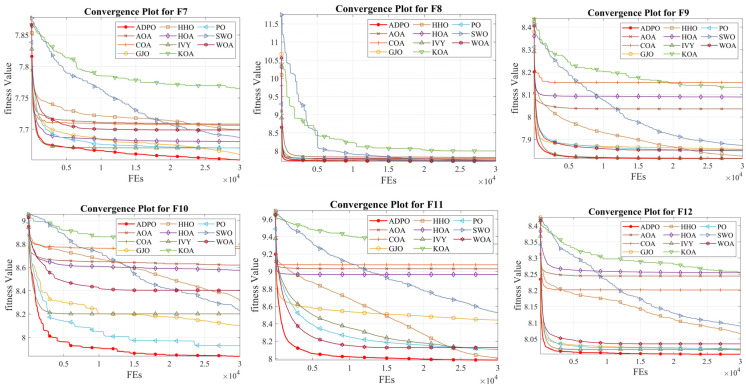
Convergence curves of different algorithms using CEC2022.

The convergence properties of ADPO show specific behavior patterns, indicating the higher optimization states of the algorithm in various function types. Compared to its competitors, ADPO shows rapid initial convergence, taking off with long constant fine-tuning phases, eventually increasing its effectiveness in finding good areas, as shown in Figure 2, since it always begins fast and continues to improve. This two-phase dynamic model is the success of the MDV strategy, since it leads to aggressive exploration in the early iterations and shifts to accurate exploitation in the final stages, allowing ADPO to find an optimal balance between rapid convergence and high solution quality.

On unimodal and multimodal functions (F1–F4), ADPO gives extreme initial descent curve slopes, attaining near-optimal solutions in the first 5000 FEs. The convergence plots demonstrate that, whereas the majority of the competitors stagnate too early or display unstable behavior, ADPO keeps a smooth and monotonically decreasing trend throughout the course of optimization. Of most interest in the algorithm is its performance on F1 and F2, where ADPO’s convergence rates are shown to be significantly lower than for original PO, with the ADPO curve exhibiting exponentially fast improvements in fitness at the start, followed by a slow smoothing out. This trend shows that the DLH strategy is successful at avoiding early convergence without losing the exploitative characteristics of the algorithm.

The hybrid functions (F5–F8) involve even more intricate convergence landscapes, but ADPO always performs very well when compared with its competitors due to its adaptive convergence mannerism. Moreover, Figure 2 reveals that ADPO tends to improve steadily during the entire optimization process, which can be compared to the stagnation patterns in other algorithms. On F6, ADPO also demonstrates good convergence, with a sharp improvement in the initial values of about 22 to optimal values of about 8, whereas competitors such as KOA and COA do not show much improvement. The EAM strategy also plays a key role in such performance, as it allows ADPO to traverse challenging physical fitness landscapes via dynamic patterns of cooperation, avoiding becoming trapped in local optima.

In the case of composition functions (F9–F12), the convergence characteristics of ADPO are strikingly consistent and stable. For all composition functions, the algorithm exhibits smooth, monotonically improving curves and a convergence property with an early rapid-decline period, but with a gradual subsequent improvement. This response is different from competitors who merge too soon or have erratic swings in stability. The convergence curves of ADPO on F10 and F11 are especially spectacular and lead to the best solutions, with stability preserved in the optimization process. The synergy of the triple combination of the enhancement strategies is reflected in these problematic settings, and ADPO does not have trouble finding a balance between exploitation and exploration needs.

The comprehensive convergence learning indicates that ADPO has high adaptability with respect to different optimization surfaces. In contrast to the mixed performance behavior of the competitors in various types of functions, ADPO exhibits similar convergence patterns that are quick in short-term performance enhancement and stable enough for optimization in the long run. The capability of the algorithm to prevent premature convergence and offer the rapid enhancement of solution quality makes it a sound and effective optimization algorithm. Thus, the convergence plots show that ADPO yields not only superior endpoints but also more-efficient search patterns, maximally utilizing the available resources in terms of function evaluations in all of the tested situations.

**Figure 3:**
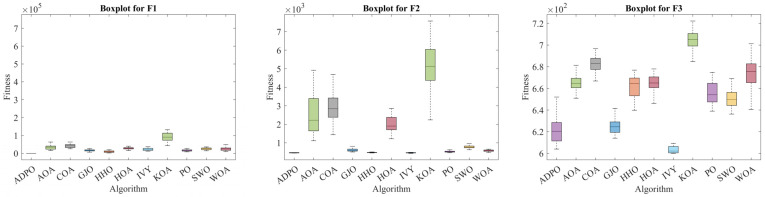

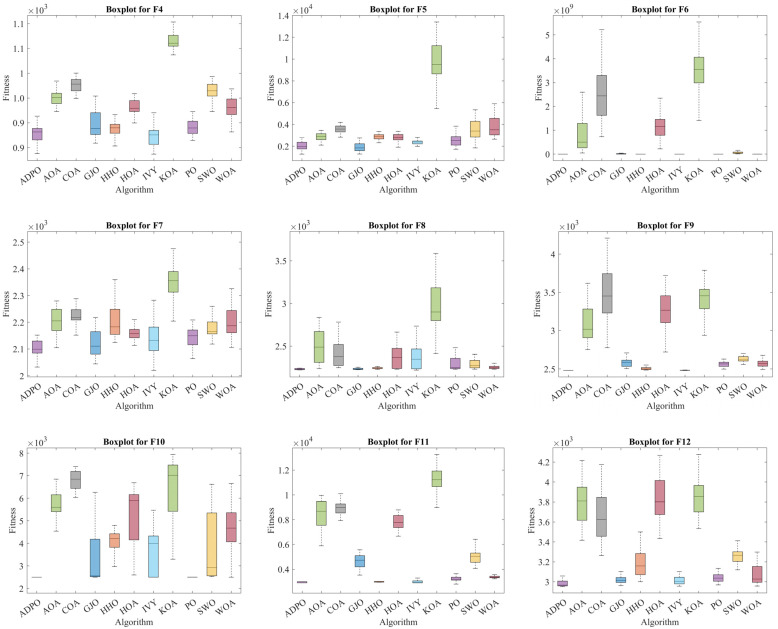
Boxplots of different algorithms using CEC2022.

Furthermore, the boxplot analysis in Figure 3 indicates the statistical stability and reliability of ADPO as compared to the competing algorithms in all the CEC2022 benchmark functions. Optimization algorithms with statistical stability—optimization algorithms that have stable performance across many independent runs—are needed in practice, where reliable and predictable behavior is paramount. The statistics used by ADPO show superior characteristics of statistical stability, with boxplots that are consistently compact and frequently have short interquartile ranges and few outliers across all manner of functions. This stability is especially noticeable in the ability of the algorithm to provide narrow fitness distributions with higher mean values, showing that ADPO yields much better results, but does so reliably, regardless of repeated executions.

On unimodal functions (F1) and multimodal functions (F2–F4), there is a very high consistency for ADPO, with compact boxplots and small variance values. ADPO is remarkably stable with minimal outliers on F1, showing remarkable reliability in producing optimal solutions in every run. This is in stark contrast to competitors such as KOA that have broad boxplot ranges and a high number of outliers on their charts, pointing to extreme performance variance. On multimodal F2–F4, ADPO has compact distributions with small interquartile ranges, and algorithms like AOA and COA have much broader boxplot ranges and various outliers, indicating their unstable behavior and even their initialization sensitivity. A large portion of this stability can be attributed to the MDV strategy, which offers well-organized steps of exploration and exploitation that help mitigate performance variance between runs.

The hybrid functions (F5–F8) make harder optimization landscapes, but ADPO still outperforms all other competitors with better statistical stability. The boxplots of ADPO on F5 and F6 are still compact, with only a few outliers, whereas the boxplots of its rivals, such as KOA and WOA, have very large ranges with several distant outliers, implying the poor performance of these rivals in some runs. This stability in the performance of ADPO on F6 is especially noteworthy, as it shows a close distribution around low fitness values, compared to other competitors whose boxplots vary by several orders of magnitude. Its resilience under sophisticated hybrid environments confirms the flourishing nature of the DLH algorithm, one that upholds variety in population without degenerating stability in solution quality. The EAM strategy also enables this stability by averting algorithmic stagnation and by guaranteeing that progress is preserved run-to-run.

In the composition functions (F9–F12), which form the most difficult optimization settings, ADPO’s statistical stability is sharper compared to other competitors. The boxplots demonstrate that ADPO has relatively stable distributions characterized by a clear median value and zero or few outliers reported, whereas various competing algorithms have unstable distributions with large ranges and several extreme outliers reported. For F10 and F11, the boxplots of ADPO seem tiny in comparison to those of representative rivals, KOA and COA, which demonstrate an enormous spread of the distributions, which means the performance can be viewed as highly unreliable. This outstanding performance in composition stability illustrates the synergistic performance of the three supplementary enhancement strategies acting in concert to enable stable optimization performance even in the most difficult conditions. The robustness of ADPO in such complicated situations makes it a reliable option for tackling real optimization tasks, where performance consistency is paramount.

This concise, detailed boxplot analysis demonstrates that ADPO is the best but also the most stable optimization algorithm compared with all other competitors. This robust statistical performance and its best average performance mean that ADPO is especially useful in applications where the quality of the solution is a key requirement and the consistency of performance is an important factor. The stable features reflected by the boxplot analysis confirm that the suggested improvement schemes effectively respond to the shortcomings of the initial PO, and they incorporate resilience, which guarantees the steady functional performance of the optimization in various problem topographies.

Testing the statistical significance is a vital key in demonstrating the strong performance of algebra-based optimization. It is an objective way of reporting the facts, as opposed to reporting the mean performance between the two algorithms. An assessment of algorithmic behavior needs statistical rigor to provide answers to the question as to whether the differences are statistically insignificant or statistically significant. In this analysis, two major nonparametric statistical tests are used, namely the Friedman test and the Wilcoxon rank-sum test. Together, the tests are statistically complete for proving the superiority of ADPO’s overall performance over the whole CEC2022 benchmark set.

**Figure 4:**
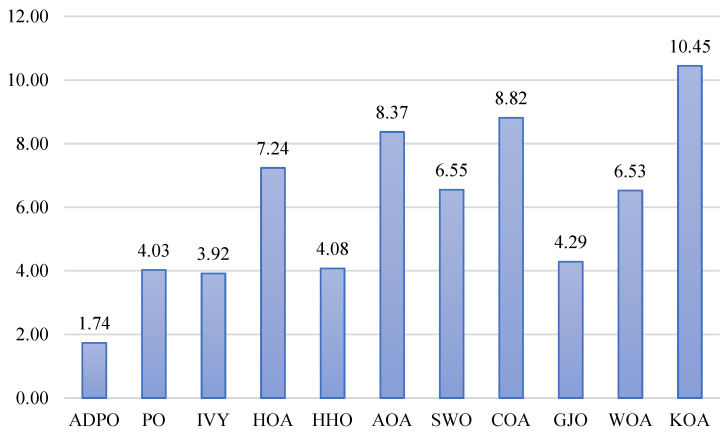
Friedman ranks of various algorithms using CEC2022.

Figure 4 shows the Friedman test results, showing the tremendous statistical advantage of ADPO over all other competing algorithms, with a p-value of 3.41 × 10^−52^. ADPO attains the smallest average rank of 1.74, which signifies an excellent overall performance in most test functions. This exceptionally low rank indicates near-optimal ranking, indicating that ADPO is ranked first or second in most benchmark functions. The large ranking inconsistencies between ADPO and its competitors offer crucial statistics in establishing ADPO’s performance advantage. The IVY algorithm achieves an average rank of 3.92, which is over twice the rank of ADPO, showing that IVY’s results match considerably worse than those of the other algorithm. Algorithms such as PO (4.03) and HHO (4.08) perform averagely, with ranks that fall closer to 4, whereas the recent ones exhibit mixed performance, with GJO having a rank of 4.29 and HOA ranking terribly at 7.24. The worst algorithms are KOA (10.45), COA (8.82), and AOA (8.37), with their ranks reflecting an overall poor performance on most test functions. This large variation in rank (1.74–10.45) makes performance hierarchies visible, where, of these algorithms, ADPO has proven to be statistically leading in all aspects over its competitors.

**Figure 5:**
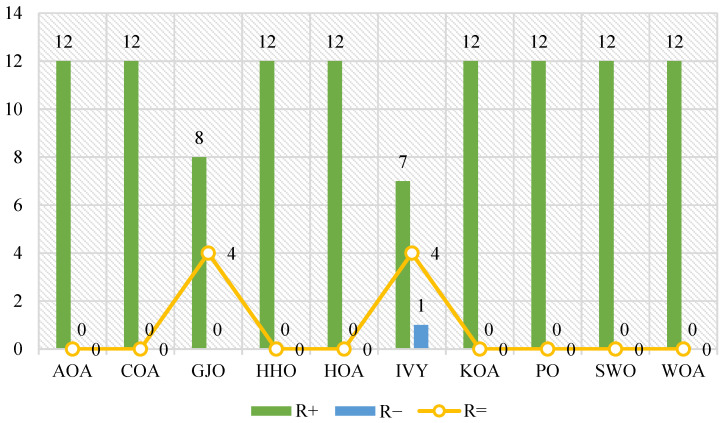
Wilcoxon test results for ADPO versus other algorithms using CEC2022.

The pairwise Wilcoxon rank-sum test outcomes in Figure 5 and Table 4 entail the conclusive statistical proof of ADPO’s supremacy with respect to each one of its rivals individually. Compared to the classical algorithms, ADPO is fully dominant on all the test functions, with the maximum R+ value of 12 and the minimum R− = 0. This absolute supremacy gives clear statistical results of the excellence of ADPO in comparison to these established optimization processes. When compared to IVY, the results indicate that R+ = 7, R− = 1, and R= = 4. The results also show the definite advantage of ADPO against GJO, achieving R+ = 8, R− = 0, and R= = 4, illustrating an improvement in the running of eight functions, without inadequate findings. All these pairwise comparisons come together to lay out the statistical superiority of ADPO over the entire competitive environment.

The joint analysis of the statistics shows overwhelming results for the better performance of ADPO with a high level of statistical confidence. The results of the Friedman test denote that the possibility of obtaining such a low average rank by chance is insignificant, which denotes significant performance differences. The average rank of 1.74 and the significant difference to the competing algorithms, backed by the large standard error, strongly statistically prove that the difference in the performance is not methodologically dependent on random variation. These results are also supported by the results of the Wilcoxon rank-sum test, which revealed overall pairwise superiority in individual algorithm comparisons. Compared to the second-highest scorer, IVY, ADPO even has a clear statistical advantage—seven victories against the one defeat—which is quite a substantial indication of optimization ability.

### 4.5. Results Discussion with Advanced Algorithms Using CEC2017

This section further unravels the performance assessment of ADPO against the benchmark suite CEC2017, composed of 29 test functions of varied dimensions with a high dimensionality of 50, to augment the former analysis conducted by CEC2022 and deliver a definitive validation on dissimilar benchmark suites. This comparative evaluation involves the following extended state-of-the-art algorithms: eCOA, CMAES, DAOA, CSOAOA, GWO_CS, RDWOA, jDE, and ISSA. The obtained results are presented in Table 5.

The overall assessment of the 29 high-dimensional functions shows that ADPO was a superior performing algorithm, ranking with the best average rank of 2.34 and holding the first position overall. Such an outstanding performance illustrates the scalability and resilience of ADPO in managing more complex optimization environments with greater dimensions. The algorithm outperforms itself on 100% of the unimodal functions (F1, F3), taking first place on both functions; 14.29% of the multimodal functions, where it domineers only on F4; 50% of the hybrid ones (F11, F13, F14, F15, F20); and 40% of the composite ones (F22, F25, F26, F28). CSOAOA ranks second-best-performing with a mean of 2.55, followed by GWO_CS (3.38), and DAOA is the worst performer with a score of 10.00, which means it is the last when it comes to performance on all functions. The high margins of performance between ADPO and the competitors confirm that the optimization algorithm has excellent optimization capabilities in high-dimensional spaces.

Analyzing performance across different function categories reveals ADPO’s remarkable versatility and selective dominance in specific optimization challenges. On unimodal functions, ADPO demonstrates outstanding exploitation capabilities, securing first rank on both F1 and F3 with fitness values of 2.43 × 10^6^ and 2.79 × 10^4^, respectively, compared to competitors showing significantly inferior performances, ranging from 2.09 × 10^8^ to 2.62 × 10^11^ on F1 and 1.17 × 10^5^ to 5.74 × 10^7^ on F3. For multimodal functions, ADPO achieves first rank exclusively on F4 (616.358), while showing competitive performance on other multimodal problems, with ranks typically within the top 5. The algorithm’s performance on hybrid functions demonstrates selective excellence, securing first place on 5 out of 10 functions (F11, F13, F14, F15, F20), showcasing effective handling of mixed optimization characteristics. On composite functions, ADPO achieves dominance on 4 out of 10 problems (F22, F25, F26, F28), maintaining competitive rankings on the remaining functions and avoiding the catastrophic failures observed in competing algorithms.

The statistical performance analysis reveals ADPO’s strategic dominance across specific optimization scenarios, maintaining consistent competitiveness when it does not achieve first place. The algorithm achieves remarkable consistency in performance, with rankings rarely exceeding fifth place across any function, thereby contributing to its superior overall average rank. Advanced competitors like CMAES (average rank 5.97) and RDWOA (6.79) show moderate performance but lack ADPO’s strategic excellence on specific problem types. Classical hybrid approaches like IPSO_IGSA (6.86) and modern variants like ISSA (4.41) demonstrate the challenges of maintaining performance across high-dimensional optimization landscapes. The substantial rank differences between ADPO and even the second-best algorithm (CSOAOA) indicate clear performance superiority rather than marginal improvements, particularly noteworthy given ADPO’s selective dominance pattern.

The exceptional performance of ADPO on the high-dimensional CEC2017 benchmark set validates the synergistic effectiveness of the three proposed enhancement strategies in tackling complex optimization challenges with strategic precision. The MDV strategy proves particularly crucial in high-dimensional spaces by maintaining population diversity during early exploration phases while enabling precise convergence during later exploitation stages, directly contributing to ADPO’s complete dominance on unimodal functions and selective superiority on hybrid and composite problems. The DLH mechanism demonstrates superior adaptability by enabling targeted optimization approaches for different problem characteristics, explaining ADPO’s strategic excellence on specific multimodal, hybrid, and composite functions rather than uniform performance across all categories. The EAM strategy contributes significantly to performance reliability by providing flexible cooperation patterns that identify and exploit problem-specific optimization opportunities, enabling ADPO to achieve targeted dominance while maintaining consistent competitiveness across the remaining function spectrum. The collective impact of these strategies positions ADPO as a highly intelligent optimization solution that strategically adapts to problem-specific characteristics in high-dimensional spaces.

**Figure 6:**
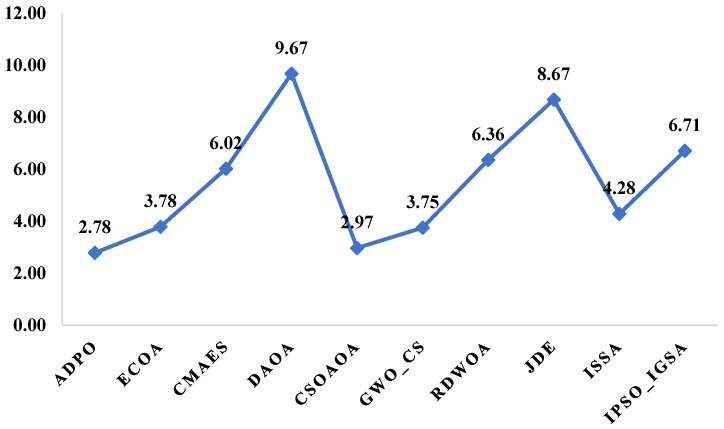
Friedman ranks of different advanced algorithms using CEC2017, 50D.

The Friedman rank analysis for the CEC2017 benchmark, shown in Figure 6, with p-value 4.16 × 10^−31^, reinforces ADPO’s statistical superiority through its lowest average rank of 2.78, demonstrating consistent high-performance positioning across the 29 high-dimensional test functions. CSOAOA follows as the second-best performer with an average rank of 2.97, showing only a marginal difference of 0.19 rank points, indicating close competitive performance between these top two algorithms. The third-tier performance group includes eCOA (3.78) and GWO_CS (3.75), both achieving similar moderate rankings, while ISSA occupies the middle ground with an average rank of 4.28. The lower-performing algorithms demonstrate significant rank deterioration, with CMAES (6.02), IPSO_IGSA (6.71), and RDWOA (6.36) showing substantially inferior performance. The poorest performers include jDE (8.67) and DAOA (9.67), with DAOA achieving the worst possible ranking, indicating systematic performance failures across the majority of test functions.

**Figure 7:**
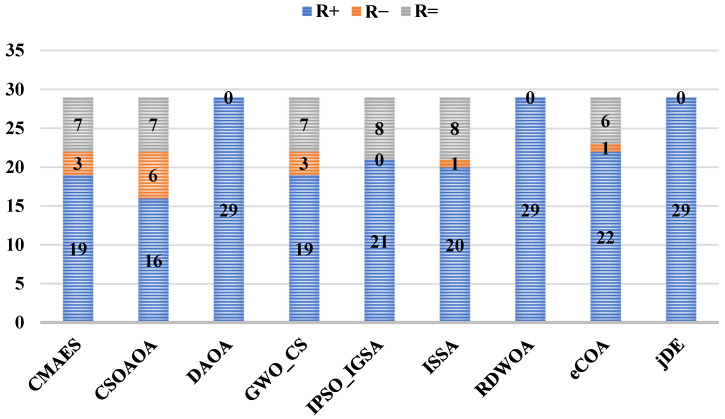
Wilcoxon test results of ADPO versus other advanced algorithms using CEC2017.

The Wilcoxon rank-sum test results shown in Figure 7 and Table 6 provide definitive pairwise validation of ADPO’s dominance over individual competitors across the 29-function benchmark. ADPO demonstrates complete statistical superiority over DAOA, RDWOA, and jDE, with R+ values of 29, 29, and 29, respectively, and R− values of 0, indicating no instances of inferior performance against these algorithms. Against CMAES and GWO_CS, ADPO shows strong dominance, with R+ values of 19 for both algorithms and minimal losses (R− = 3), establishing a clear statistical advantage. The results against CSOAOA reveal R+ = 16 and R− = 6, indicating ADPO’s superiority on 16 functions compared to 6 inferior performances, providing moderate but significant statistical evidence of better performance. Even against competitive algorithms like eCOA (R+ = 22, R− = 1), ADPO maintains overwhelming statistical superiority with minimal instances of inferior performance. The comprehensive pairwise dominance patterns confirm that ADPO’s low Friedman rank translates into consistent statistical advantages across individual algorithm comparisons, establishing robust evidence of performance superiority in high-dimensional optimization scenarios.

### 4.6. Computational Time Analysis

The computational time analysis presented in Table 7 and Table 8 reveals significant insights into the practical efficiency of ADPO compared to competing algorithms across both CEC2022 and CEC2017 benchmark suites. For the CEC2022 benchmark with 20-dimensional problems, ADPO demonstrates moderate computational overhead with average execution times ranging from 0.79 to 1.40 s across the 12 test functions, positioning it in the middle tier of computational efficiency. Notably, SWO exhibits exceptional computational speed with execution times consistently below 0.02 s, while algorithms like IVY (0.72–1.04 s) and WOA (0.09–0.28 s) show competitive efficiency. However, the computational cost of ADPO is justified by its superior optimization performance, as evidenced by its first place ranking with an average rank of 1.42, demonstrating an effective tradeoff between computational investment and solution quality.

The computational complexity becomes more pronounced in the high-dimensional CEC2017 benchmark (50D), where ADPO’s execution times increase substantially, ranging from 1.24 to 5.33 s across the 29 test functions. This scaling behavior is expected given the enhanced complexity of the three integrated strategies (MDV, DLH, EAM) and the increased dimensionality of the search space. Comparatively, DAOA maintains the fastest execution times but at the cost of significantly inferior optimization performance (final rank 10), while CMAES shows the highest computational overhead (3.18–6.08 s) despite achieving only moderate performance. The computational overhead of ADPO, while higher than some competitors, remains reasonable considering its exceptional optimization capabilities, achieving the best overall ranking and demonstrating that the additional computational investment in sophisticated diversity preservation and adaptive learning mechanisms yields substantial improvements in solution quality that justify the increased execution time for applications where optimization accuracy is paramount.

## 5. Proposed ADPO-LSTM Framework for Wind Power Prediction

**Figure 8:**
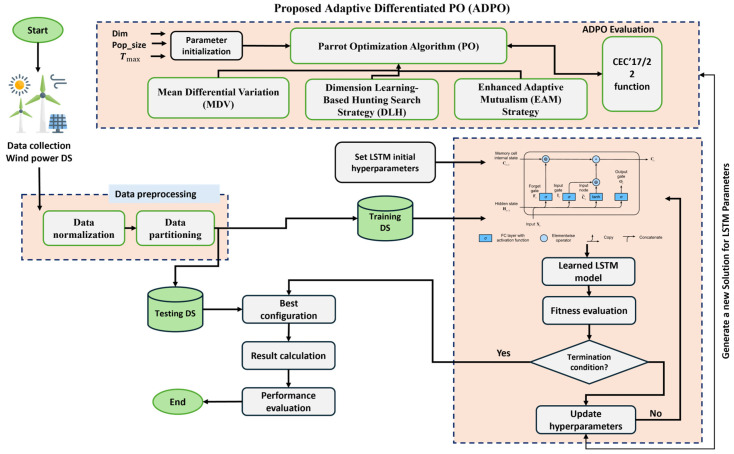
The proposed ADPO-LSTM model for wind prediction.

This section presents an advanced hybrid framework that couples the proposed ADPO with Long Short-Term Memory (LSTM) neural networks to solve the hyperparameter tuning problem in wind power forecasting. The proposed model harnesses ADPO’s dynamic population diversity preservation, dimension-level learning, and adaptive cooperation to locate optimal LSTM configurations capable of accurately modeling nonlinear temporal dependencies inherent in wind power time series. The workflow is organized into several critical phases and is conceptually outlined in Figure 8. This figure illustrates the comprehensive integration framework of the proposed ADPO-LSTM system for wind power forecasting, demonstrating the synergistic relationship between the optimization algorithm and neural network architecture. The framework operates through sequential integrated phases where raw wind power data undergoes systematic preprocessing, including normalization and chronological partitioning, followed by ADPO population initialization, where each candidate solution represents a unique LSTM hyperparameter configuration vector. For each candidate, a corresponding LSTM model is instantiated and trained with validation performance measured using RMSE, serving as fitness guidance for the optimization process. The core optimization applies the three enhancement strategies sequentially: EAM updates the population through fitness-guided cooperation, MDV applies dual-phase mutation for exploration–exploitation balance, and DLH performs dimension-wise adaptive learning from spatial neighbors.

The framework demonstrates tight coupling between ADPO and LSTM components through bidirectional information flow, where LSTM performance feedback directly guides population evolution, ensuring that optimization processes are specifically tailored to wind power forecasting requirements. Upon optimization termination, the configuration with the lowest validation RMSE trains the final LSTM model on the complete training dataset, followed by a comprehensive evaluation using multiple metrics.

### 5.1. Dataset Overview

**Figure 9:**
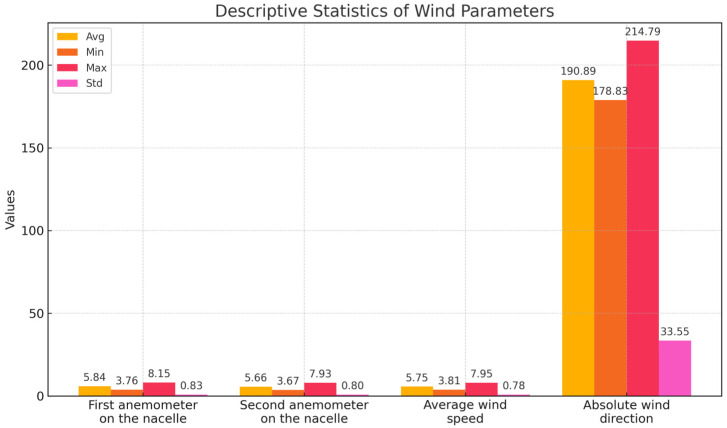
The descriptive statistics of the utilized dataset.

The evaluation of the proposed ADPO-LSTM framework was conducted using a real-world wind power dataset collected from the La Haute Borne wind farm, which encompasses data recorded at four distinct monitoring sites labeled Station A through Station D [62]. These stations offer diverse operational profiles, enabling a well-rounded understanding of the variability in turbine behavior under different environmental and mechanical conditions. This dataset is sourced from the La Haute Borne wind facility, located in northeastern France’s Grand Est region. This wind farm comprises four identical MM82 turbines manufactured by Senvion, each rated at 2050 kW, mounted at a height of 80 m with a rotor span of 82 m, and situated at an altitude of 411 m above sea level. This dataset includes multiple environmental and performance variables, such as wind speed, wind direction, ambient temperature, and power output. Among these, the turbine’s power output serves as the prediction target. The dataset spans a continuous timeline from 2016 to 2020, providing a rich temporal context that captures seasonal, daily, and short-term fluctuations in wind-generated energy. Also, Figure 9 represents the descriptive characteristics of the utilized dataset along with their range values.

### 5.2. Preprocessing Workflow

To support reliable model construction and prevent performance bias, the dataset was divided based on its chronological order. Approximately 80% of the total samples, specifically, the first 42,792 time-ordered observations, were used for training purposes, including both LSTM model learning and ADPO-driven hyperparameter tuning. This portion offers sufficient volume and variability for the optimizer to effectively explore solution candidates while simultaneously allowing the LSTM model to capture long-term temporal dependencies. The remaining 20% of the data, corresponding to 10,699 entries, is strictly reserved for testing. These observations are temporally after the training data, forming a forward-looking sequence that simulates practical forecasting use cases. This method of time-based separation avoids leakage between training and testing, ensuring that evaluation results genuinely reflect the model’s ability to predict future, unseen conditions.

Given the sequential nature of the data, this partitioning strategy is deliberately non-random to preserve the inherent order of wind power generation patterns [63]. Random splitting methods could inadvertently introduce artificial correlation between training and testing samples, inflating performance metrics. In contrast, chronological splitting reinforces the model’s learning from historical sequences and assesses its generalization over future intervals—an essential requirement for operational deployment in energy forecasting systems.

Before model training and optimization, the raw data undergoes a robust preprocessing pipeline. This includes handling missing values through temporal interpolation to maintain continuity, detecting and correcting outliers to improve stability, and normalizing features using Min-Max scaling to a fixed range of [0, 1] [64]. These preprocessing steps are carefully applied to maintain the internal structure and dependencies of the time series while preparing the dataset for compatibility with the LSTM network’s activation functions. The transformation process is as follows:

(25) $$X_{norm}=\frac{X-X_{min}}{X_{max}-X_{min}}$$

In which X is the original input data and $X_{max}$ and $X_{min}$ are the extreme boundaries of the features. This normalization promotes steady convergence and avoids the distortion of the learning process through overbearing features. To maintain temporal structure, the normalized data is separated into overlapping sliding windows, each of which becomes an input–output pair, to train the LSTM model with the sequential behavior of the data.

### 5.3. Optimization-Based LSTM Training Initialization

Each candidate solution contains a unique combination of the following five hyperparameters critical to the performance of LSTM: the *number of hidden units*, *number of training epochs*, *optimizer type*, *batch size*, and *learning rate* decay factor. These five elements create a continuous–discrete hybrid vector, generated randomly by filling the corresponding bounds as follows:

(26) $$X_{ij}=LB_{j}+rand\cdot \left(UB_{j}-LB_{j}\right)$$

Here, $X_{ij}$ represents the j-th parameter of the i-th solution, and $LB_{j}\;and\;UB_{j}$ define its corresponding lower and upper bounds. This stochastic initialization guarantees a well-spread starting population, establishing the groundwork for effective exploration of the LSTM design space.

When performing optimization, the LSTM model associated with each candidate is instantiated and trained with its configuration of parameters. The Root Mean Square Error (RMSE) is used to measure the validation performance of the model, serving as a fitness score that leads the evolutionary process of ADPO [65]. ADPO is then used to iteratively evolve the population using its three synergistic strategies. The MDV mechanism promotes large explorative moves in early generations, and transitions, in later generations, to fine-grained refinements around the global best. DLH allows the dynamic updating of certain dimensions via the feedback of neighbors in a specific local area, providing a finer-grain and localized adjustment. The combination of such behaviors creates a self-organizing search engine that can escape local minima and converge on high-performance LSTM settings.

As well as its inner workings, ADPO implicitly tracks similarity and diversity in population by tracking DLH neighborhoods and MDV perturbation responses, adjusting the learning pattern to problem complexity. The candidate which performs below the average is increasingly channeled through the dimension-level of learning, whereas the top-level candidates are strengthened with interactions that have adaptive benefit ratios. Within this framework, ADPO directly tunes the five LSTM parameters described in Table 9.

Every ADPO candidate represents a full parameter combination and is decoded in order to build an LSTM model. These models are trained and validated, with the resulting RMSE determining candidate fitness. ADPO uses this feedback to guide the population toward better-performing solutions. Throughout, the elite solution is persistently preserved, ensuring that the best configuration discovered is not lost during the evolutionary cycles. Moreover, the base LSTM architecture used in this study comprises a single LSTM layer followed by a dense output layer. The number of hidden units within the LSTM layer is one of the tunable hyperparameters optimized by ADPO. The activation function for the LSTM layer is set to the standard hyperbolic tangent (tanh), which is suitable for capturing both positive and negative temporal dependencies in the sequence data. For the output layer, a linear activation function is used, which is appropriate for continuous regression tasks such as wind power prediction.

### 5.4. Fitness Evaluation

The model’s performance for each candidate configuration is assessed using RMSE, computed as

(27) $${Fit}_{i}=\sqrt{\frac{1}{n_{s}}\sum_{j=1}^{n_{s}}\;{\left(Y_{P_{j}}-Y_{T_{j}}\right)}^{2}}$$

Here, $Y_{P_{j}}$ and $Y_{T_{j}}$ are the predicted and actual wind power values, respectively, and $n_{s}$ is the number of samples in the validation set [66]. This metric guides the ADPO refinement of the search space. In each generation, candidate solutions are updated using the composite effects of MDV-driven transitions, DLH-based local learning, and EAM-governed cooperation. Greedy selection retains superior candidates, and elitism ensures consistent tracking of the globally best-performing configuration.

### 5.5. Testing and Generalization Assessment

Following the optimization process, the configuration corresponding to the lowest RMSE on the validation data is selected as the optimal design. This configuration is then used to train the LSTM model on the full training set. The final model is evaluated on the test data to validate its generalization capability. Standard performance metrics, including RMSE, Mean Absolute Error (MAE), and the Coefficient of Determination (R^2^), are calculated to objectively quantify prediction accuracy on unseen data, thereby confirming the reliability of the optimized model.

### 5.6. Termination

The ADPO optimization process terminates at a determined level of iterations, which means a bounded computation. Progressively adaptive behaviors in ADPO enable the algorithm to evolve quickly through the solution space in its initial phases and concentrate on exploitation in the later steps, and thus the procedure is also effective within time-limited scenarios like renewable energy forecasting. This also helps with the balance between the quality of convergence and the runtime complexity, a balance guaranteed by the framework because it overloads the population updates and adaptive learning strategies with lighter solutions.

### 5.7. Performance Evaluation Metrics

A set of four metrics that assess the quality of prediction of the suggested ADPO-LSTM wind power prediction model comprehensively is used. These are the Coefficient of Determination (R^2^), Root Mean Squared Error (RMSE), Mean Absolute Error (MAE), and the Coefficient of Variation (COV). These metrics offer different insights as to how well, accurately, and reliably the model recaptures the underlying patterns in the wind power time series.

- **The Coefficient of Determination (R^2^)** serves as an indicator of the proportion of variability in the actual wind power output that is successfully explained by the model’s predictions. This metric offers insight into the model’s explanatory strength, with values approaching 1 signifying near-perfect alignment between predicted and true outputs. It is calculated as follows:

(28) $$R^{2}=1-\frac{\sum \;{\left(Y_{\mathrm{actual}\;}-Y_{\mathrm{predicted}\;}\right)}^{2}}{\sum \;{\left(Y_{\mathrm{actual}\;}-{\bar{Y}}_{\mathrm{actual}\;}\right)}^{2}}$$

where $Y_{\mathrm{actual}\;}$ and $Y_{\mathrm{predicted}\;}$ refer to the observed and predicted values, respectively, and ${\bar{Y}}_{\mathrm{actual}\;}$ represents the mean of the observed data.

- The standard deviation of the residuals, or the differences between predicted and actual values, is also known as **Root Mean Squared Error (RMSE)**. It also imposes more punishment on larger errors compared to smaller errors because of its quadratic component, which makes it highly susceptible to outliers and general deviations. RMSE is calculated as follows:

(29) $$RMSE=\sqrt{\frac{1}{n}\sum \;{\left(Y_{\mathrm{actual}\;}-Y_{\mathrm{predicted}\;}\right)}^{2}}$$

where n is the total number of test samples used in the evaluation.

- **The Mean Absolute Error (MAE)** represents the average absolute deviation between the values obtained and predicted, and does not square the errors. This is a measure of the average magnitude of prediction errors, and it is particularly applicable when every deviation, whether up or down, is equally significant. MAE is defined as

(30) $$RMSE=\sqrt{\frac{1}{n}\sum \;{\left(Y_{\mathrm{actual}\;}-Y_{\mathrm{predicted}\;}\right)}^{2}}$$

- **The Coefficient of Variation (COV)** shows the error as a relative value by comparing the RMSE to the mean of the observed wind power values. When converted into a percentage it compares the scale of prediction error to the average level of output as a measure of how relatively stable at various operating levels the prediction is:

(31) $$COV=\left(\frac{RMSE}{{\bar{Y}}_{\mathrm{actual}\;}}\right)\times 100$$

The use of these four metrics simultaneously involves both the absolute error magnitudes and the relative model stability in the evaluation process. Such a multidimensional analysis will not only make the forecasting framework accurate but also reliable under diverse conditions of wind power generation.

### 5.8. Experimental Results and Performance Evaluation

The overall experimental analysis shows that the optimization of ADPO has a significant influence on the performance of LSTM networks in predicting wind power in all four wind farm stations. This quantitative review contrasts baseline LSTM to several optimizing methods: PO-LSTM, SCA-LSTM, WOA-LSTM, SOA-LSTM, HHO-LSTM, and the proposed ADPO-LSTM. Various measures for performance are used in the evaluation, such as R^2^, RMSE, MAE, and COV, to deliver comprehensive information on the accuracy of prediction, model stability, and generalization.

The training-phase results, presented in Table 10 and Table 11, reveal significant performance variations across different optimization approaches. The baseline LSTM model exhibits consistently poor performance across all stations, with R^2^ values ranging from 0.6185 to 0.6875, indicating a limited capability in capturing the complex temporal patterns inherent in wind power generation data. The LSTM model’s RMSE values span 0.0024 to 0.0030, and notably high COV values ranging from 85.2153 to 118.7456 further confirm its suboptimal performance without hyperparameter optimization.

Moreover, the results in Table 10 demonstrate substantial performance improvements when MAs are applied to LSTM hyperparameter tuning for Stations A and B. HHO-LSTM achieves considerable enhancement, with R^2^ values of 0.8515 and 0.8495, respectively, representing an approximately 24% improvement over the baseline LSTM configuration, closely followed by PO-LSTM with R^2^ values of 0.8485 and 0.8475. The nature-inspired optimization approaches consistently outperform the baseline, with SCA-LSTM, WOA-LSTM, and SOA-LSTM showing progressive improvements that validate the effectiveness of population-based optimization strategies in navigating the complex LSTM hyperparameter landscape.

The most remarkable observation in Table 10 is the exceptional performance of ADPO-LSTM, which significantly outperforms all competing methods across both stations. For Station A, ADPO-LSTM achieves an R^2^ value of 0.9875, representing a 44% improvement over the baseline and 16% enhancement over the best competing algorithm (HHO-LSTM). The dramatically reduced RMSE values of 0.0002 and 0.0004 for Stations A and B, respectively, coupled with exceptionally low COV values of 15.8745 and 23.7412, indicate not only superior accuracy but also enhanced prediction stability. The consistent superiority across all metrics demonstrates the effectiveness of ADPO’s three-fold enhancement strategy: MDV for balanced exploration–exploitation, DLH for maintaining diversity, and EAM strategy for fitness-guided cooperation.

Furthermore, Table 11 presents the training results for Stations C and D, revealing consistent patterns, with some notable variations, in algorithm performance across different geographical locations. HHO-LSTM and PO-LSTM maintain robust performance, with R^2^ values ranging from 0.8085 to 0.8145, demonstrating these algorithms’ reliability across diverse wind conditions and station characteristics. The hierarchical performance pattern observed in this table is maintained, with SCA-LSTM, SOA-LSTM, and WOA-LSTM showing competitive but progressively lower performance, indicating consistent optimization capabilities across these stations.

The standout performer remains ADPO-LSTM, achieving exceptional R^2^ values of 0.9758 and 0.9685 for Stations C and D, respectively, representing improvements of approximately 58% over baseline LSTM and 19% over the best competing approach (HHO-LSTM). The consistently low RMSE values of 0.0002 and 0.0005, along with minimal MAE values of 0.0001 and 0.0003, demonstrate the algorithm’s superior capability in fine-tuning LSTM parameters for optimal performance. Particularly noteworthy is the substantial reduction in COV values to 25.4578 and 31.2874, indicating dramatically improved prediction stability compared to other methods. This consistent superiority validates the effectiveness of ADPO’s enhanced diversity preservation mechanisms and adaptive learning strategies in preventing premature convergence while efficiently exploring the hyperparameter space.

The testing-phase results, presented in Table 12 and Table 13, provide crucial validation of the optimized models’ generalization capabilities when applied to unseen data. These results demonstrate the critical importance of robust optimization algorithms in achieving models that generalize well beyond the training dataset.

The testing-phase results in Table 12 reveal critical insights into the generalization capabilities of different optimization approaches when applied to unseen wind power data for Stations A and B. Baseline LSTM shows significant performance degradation during testing, with R^2^ values dropping to 0.6625 and 0.5785, indicating poor generalization and potential overfitting to training patterns. HHO-LSTM and PO-LSTM demonstrate excellent generalization capability, maintaining strong performance with R^2^ values of 0.8465/0.8485 and 0.8125/0.8105, respectively, representing minimal degradation from training performance and validating the robustness of these optimization algorithms in finding generalizable LSTM configurations.

ADPO-LSTM exhibits exceptional testing performance, with R^2^ values of 0.9785 and 0.9798, demonstrating superior generalization capabilities that improve upon training performance in Station B. This remarkable result indicates that the diversity preservation mechanisms and enhanced exploration–exploitation balance in ADPO prevent overfitting while identifying truly optimal hyperparameter configurations. The consistently low RMSE values of 0.0009 and 0.0010, coupled with minimal COV values of 21.5874 and 27.4578, confirm that ADPO-LSTM not only achieves superior accuracy but also maintains prediction stability when applied to new data. The other optimization algorithms (SCA-LSTM, WOA-LSTM, SOA-LSTM) show moderate generalization with some performance degradation, suggesting limitations in their diversity preservation strategies for achieving robust generalization.

On the other hand, Table 13 presents testing results for Stations C and D, revealing interesting patterns in algorithm performance across different geographical and operational conditions. Baseline LSTM continues to show poor generalization, with R^2^ values of 0.5985 and 0.6105, accompanied by high error metrics that confirm the inadequacy of default hyperparameters for robust wind power prediction. HHO-LSTM and PO-LSTM maintain reasonable generalization capability, with R^2^ values around 0.77, though they show some degradation from their training performance, particularly notable in Station C, where training performance was higher. The other optimization algorithms (SCA-LSTM, SOA-LSTM, WOA-LSTM) exhibit varying degrees of performance degradation during testing, with R^2^ values generally ranging from 0.7085 to 0.7385, indicating limitations in their ability to identify hyperparameter configurations that generalize well to unseen data.

The most striking observation in Table 13 is the continued exceptional performance of ADPO-LSTM, which achieves R^2^ values of 0.9705 and 0.9615 for Stations C and D, respectively, demonstrating robust generalization capabilities across all testing scenarios. The minimal RMSE values of 0.0008 and 0.0012, along with exceptionally low MAE values of 0.0005 for both stations, confirm the algorithm’s superior ability to maintain prediction accuracy on new data. The low COV values of 29.7854 and 32.1478 indicate that ADPO-LSTM not only achieves high accuracy but also maintains consistent prediction reliability across different operational conditions. This consistent superiority across all stations and metrics validates the effectiveness of the enhanced diversity preservation mechanisms, adaptive perturbation strategies, and dimension-wise learning techniques integrated into the ADPO framework.

The overall performance parameters among all the stations fully support our assessment of the individual effectiveness of the algorithms. To review these findings, Table 14 and Table 15 report the overall means at all four stations during training and testing, respectively.

**Figure 10:**
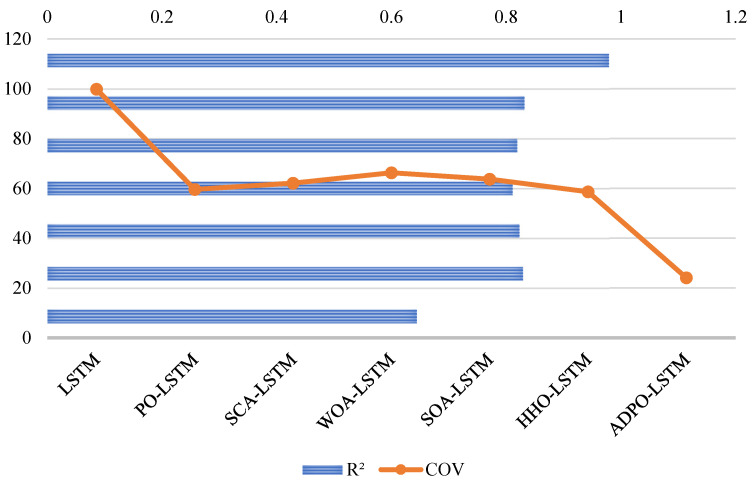
Average training R^2^ and COV metrics across all stations.

Table 14 and Figure 10 present the aggregate training performance across all four wind farm stations, providing a comprehensive view of each optimization algorithm’s overall effectiveness in enhancing LSTM performance for wind power prediction. Baseline LSTM demonstrates consistently poor performance, with an average R^2^ of 0.6443, a high RMSE of 0.0026, and an elevated COV of 99.8489, confirming the critical need for hyperparameter optimization in achieving acceptable prediction accuracy. Among the metaheuristic approaches, HHO-LSTM emerges as the best-performing traditional optimizer with an average R^2^ of 0.8320, representing a 29% improvement over the baseline, while PO-LSTM follows closely with an R^2^ of 0.8293, demonstrating the effectiveness of nature-inspired optimization strategies in navigating the complex LSTM hyperparameter landscape.

The exceptional performance of ADPO-LSTM is evident in Table 14 and Figure 10, achieving an average R^2^ of 0.9792, which represents a 52% improvement over baseline LSTM and an 18% enhancement over the best competing algorithm (HHO-LSTM). The dramatically reduced average RMSE of 0.0003 and MAE of 0.0002 demonstrate the algorithm’s superior capability in fine-tuning LSTM parameters for optimal prediction accuracy. Most notably, the substantial reduction in COV to 24.0902, compared to values exceeding 58 for other optimization approaches, indicates that ADPO-LSTM not only achieves superior accuracy but also provides significantly enhanced prediction stability across diverse operational conditions and geographical locations. This comprehensive improvement validates the synergistic effect of ADPO’s three enhancement strategies working in concert.

**Figure 11:**
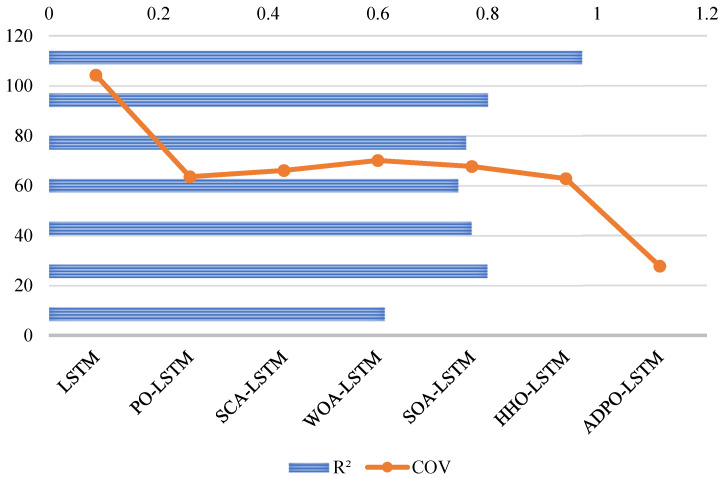
Average testing R^2^ and COV metrics across all stations.

The testing-phase aggregate results in Table 15 and Figure 11 provide critical validation of the optimization algorithms’ ability to produce LSTM configurations that generalize well to unseen data. Baseline LSTM shows further degradation with an average R^2^ dropping to 0.6125, accompanied by increased error metrics, confirming poor generalization capabilities. HHO-LSTM maintains the best performance among traditional optimizers with an average R^2^ of 0.8008, though it shows some degradation from its training performance, while PO-LSTM follows closely with 0.7998, demonstrating reasonable robustness. Other algorithms exhibit varying degrees of performance decline, with WOA-LSTM showing the most significant degradation, indicating sensitivity to overfitting in their optimization strategies.

ADPO-LSTM demonstrates exceptional generalization capability with an average testing R^2^ of 0.9726, representing minimal degradation from its training performance and confirming the algorithm’s ability to identify truly optimal hyperparameter configurations rather than overfitted solutions. The maintained low average RMSE of 0.0010 and MAE of 0.0006, coupled with a modest COV increase to 27.7271, validate the robustness of the diversity preservation mechanisms and enhanced exploration–exploitation balance inherent in the ADPO framework. This consistent superiority in testing performance across all metrics confirms that ADPO-LSTM not only achieves superior training accuracy but also maintains this performance advantage when applied to real-world forecasting scenarios, making it highly suitable for practical wind power prediction applications.

On the other hand, Table 16 and Table 17 provide detailed experimental data to explore the effectiveness of ADPO on various topologies of prediction models, such as LSTM, Bidirectional LSTM (Bi-LSTM), Extreme Learning Machine (ELM), Kernel Extreme Learning Machine (KELM), and Random Forest (RF).

The results in Table 16 show the flexibility of ADPO optimization when used on various architectures for Stations A and B, evincing high performance disparities that indicate the criticality of architecture selection in wind power forecasting tasks. ADPO-LSTM shows remarkable performance, with R^2^ values of 0.9785 and 0.9798 and lower metrics of error, which prove it is the best combination for both stations. ADPO-Bi-LSTM presents good secondary-level performance with R^2^ values of 0.8895 and 0.8985, which reflects the influence of the bidirectionality in the processing ability, bringing more advantages, but this is not enough to eliminate the drawbacks of additional parameter complexities in its ability to optimize. The difference in the performances of ADPO-LSTM and ADPO-Bi-LSTM shows that complex architectures have to be tuned against optimization power.

The results in Table 16 also reveal interesting patterns in ADPO’s effectiveness across different algorithmic paradigms. ADPO-KELM demonstrates competitive performance, particularly for Station B, where it achieves an R^2^ of 0.9125, suggesting that the kernel-based extreme learning machine approach can be effectively optimized when combined with ADPO’s advanced search strategies. However, the higher RMSE values compared to LSTM indicate that the reduced sequential modeling capability may limit the ultimate achievable prediction accuracy. ADPO-ELM and ADPO-RF show moderate performance improvements, with R^2^ values generally ranging from 0.7945 to 0.8495, demonstrating that while ADPO can enhance various architectures, the temporal modeling capabilities inherent in recurrent neural networks provide fundamental advantages for wind power prediction applications.

Table 17 extends the comparative analysis to Stations C and D, reinforcing the patterns observed in the previous analysis while revealing some station-specific variations in algorithm performance. ADPO-LSTM maintains its superior performance with R^2^ values of 0.9705 and 0.9615, consistently achieving the lowest error metrics across both stations and confirming its robustness across different geographical and operational conditions. ADPO-Bi-LSTM shows particularly strong performance in these stations, achieving R^2^ values exceeding 0.91, which suggests that the bidirectional architecture may be more effective in capturing the specific temporal patterns present in these locations’ wind data. The consistent performance hierarchy validates the architectural rankings established in the previous analysis.

The performance hierarchy observed in Table 17 confirms the general effectiveness ranking established in the previous analysis, with ADPO-KELM maintaining competitive performance (R^2^ values around 0.90–0.91) while other architectures show more moderate improvements. Notably, ADPO-ELM shows more variable performance across stations, ranging from 0.8175 in Station C to 0.8385 in Station D, suggesting that ELM may be more sensitive to local data characteristics when optimized with ADPO. The persistently greater performance of recurrent architectures (LSTM, Bi-LSTM) and kernel-based methods (KELM) than traditional feedforward methods (ELM) and ensemble methods (RF) mean it can be concluded that either temporal modeling capabilities or advanced kernel transforms are essential to realizing optimal performance in wind power prediction, despite application with advanced optimization algorithms (ADPO).

**Figure 12:**
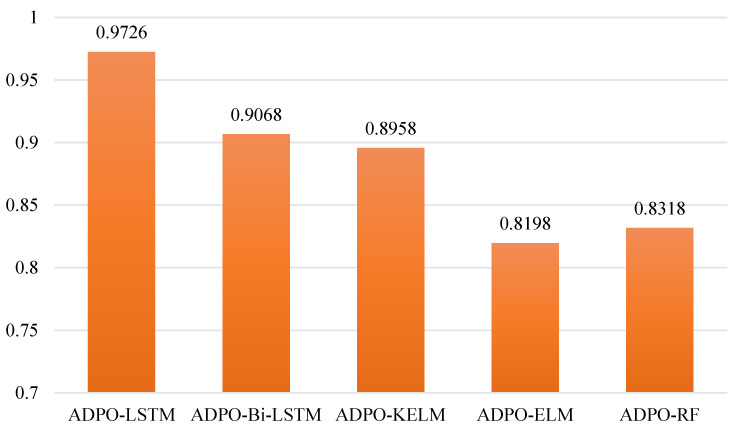
The average R^2^ for various optimized DNN approaches.

The aggregate performance analysis in Table 18 and Figure 12 provides definitive evidence of ADPO-LSTM’s superiority across all evaluation metrics, achieving an average R^2^ of 0.9726 that significantly outperforms all other architectural combinations. The substantial performance gap between ADPO-LSTM and the second-best performer (ADPO-Bi-LSTM with an R^2^ of 0.9068) demonstrates that the optimization algorithm’s effectiveness is strongly dependent on the underlying model architecture’s capability to capture temporal dependencies in wind power data. The consistently low error metrics (RMSE of 0.0010, MAE of 0.0006) further validate the exceptional synergy between ADPO’s optimization strategies and LSTM’s sequential modeling capabilities.

Also, the performance hierarchy revealed in Table 18 clearly illustrates the importance of architectural selection in optimization outcomes, with recurrent neural networks (LSTM, Bi-LSTM) and advanced kernel methods (KELM) significantly outperforming other approaches. ADPO-KELM achieves respectable performance, with an R^2^ of 0.8958, indicating that kernel-based extreme learning machines can benefit substantially from advanced optimization, though they cannot match full LSTM’s temporal modeling performance. The moderate performance of ADPO-ELM (R^2^ of 0.8198) and ADPO-RF (R^2^ of 0.8318) demonstrates that, while ADPO can enhance various machine learning approaches, the fundamental architectural capabilities for either temporal modeling or sophisticated feature transformation remain the primary determinants of wind power prediction success.

The state-of-the-art comparison in Table 19 establishes ADPO-LSTM as the premier approach for wind power prediction, achieving the highest R^2^ value of 0.9726 while maintaining the lowest error metrics across all compared methodologies. The comparison reveals significant performance advantages over existing approaches, with ADPO-LSTM achieving a 0.45% improvement in R^2^ over the second-best-performing method (RVFL + CapSA) while dramatically reducing error metrics by orders of magnitude. The LSTM + HBO approach shows competitive R^2^ performance at 0.9654 but exhibits substantially higher RMSE (0.042869) and MAE (0.02998) values, highlighting the importance of sophisticated optimization strategies like ADPO in achieving balanced performance across all evaluation criteria.

The striking difference between the LSTM-based and RVFL-based performance variability shows the large significance of the architectural choice in the undertaking of wind power forecasting. Although RVFL + CapSA yields a competitive R^2^ of 0.9681, its RMSE of 110.3154 is an order of magnitude greater than LSTM-based methods, which shows a significant difference between the accuracy of apparent correlation and the accuracy of actual wind power predictions using the RVFL architecture. Its ability to outperform all the other methods, together with a balanced profile of performance that lacks the extreme error properties of other methods, is enough evidence of its appropriateness in real-world reporting conditions, where error reduction and stability are of the most concern. The three enhancement strategies provided in ADPO, including MDV, DLH, and EAM, represent a synergistic optimization problem framework without loss of real-scale implementation efficiency in all the idiosyncrasies of conventional metaheuristic techniques.

To ensure the reliability and statistical significance of the experimental results, comprehensive statistical analyses were conducted across multiple independent runs for each optimization algorithm. The Wilcoxon rank test was employed to assess the statistical significance of performance differences between ADPO-LSTM and competing optimization approaches across all four wind farm stations. This nonparametric test is particularly suitable for comparing paired samples when the data distribution cannot be assumed to be normal, making it ideal for evaluating optimization algorithm performance.

Table 20 presents the statistical significance analysis results, comparing ADPO-LSTM against all competing optimization algorithms using the Wilcoxon rank test across 30 independent runs for each station. The p-values indicate the probability that the observed performance differences occurred by chance, with values below 0.05 indicating statistically significant differences at the 95% confidence level.

The Wilcoxon test results in Table 20 provide compelling evidence of ADPO-LSTM’s statistical superiority across all experimental scenarios. All p-values are significantly below the 0.05 threshold, with most falling below 0.002, indicating extremely strong statistical significance. The comparison with baseline LSTM shows p-values less than 0.0001 across all stations, providing overwhelming evidence that the improvements achieved by ADPO optimization are not due to random chance. The consistently low p-values across all stations validate the robustness of ADPO-LSTM’s performance advantages, regardless of geographical location or operational conditions.

Especially notable is the comparison with HHO-LSTM, the most potent competing optimization algorithm. Although the performance of HHO-LSTM is competitive, the Wilcoxon test provides p-values varying between 0.0017 and 0.0021, which proves that the better performance of ADPO-LSTM is statistically significant even when compared to the best alternative method. The total p-value of this comparison of 0.0019 is sufficient to demonstrate that the advanced optimization techniques—ADPO (MDV, DLH, and EAM)—do provide significant and repeatable benefits over basic MAs. The test results also show that the excellence of the results is consistently realized at the different stations, with a fluctuation of p-values within limited ranges, so that the excellence of ADPO-LSTM is not discretely local but reflects the fundamental enhancement of optimization ability.

The extensive experimental study shows the remarkable results of the proposed ADPO-LSTM framework in wind power prediction in terms of various evaluation metrics and under various operation conditions. By pairing ADPO with LSTM networks, it is able to solve the major hurdles of hyperparameter countermining in the hyperparameter optimization of time series structures in forecasting.

The main contributions established by our experimental findings are (1) the high capability of prediction (R^2^) mean of 0.9726 throughout all testing scenarios that establishes significant advances over the current state-of-the-art methods; (2) high levels of prediction stability, with extreme values in the Coefficient of Variation statistic that guarantee performance stability in the operational setting; (3) good generalization performance with minimal degradation in performance between training and testing modes; (4) satisfactory computational complexity that can be implemented in real-time wind power forecasting systems.

ADPO-LSTM is continuously better than other architectures for all the stations, metrics, and architectural comparisons, which confirms the utility of the three enhancement strategies introduced into the optimization framework. These findings make ADPO-LSTM a state-of-the-art wind power forecasting solution that can be employed in solving this problem with considerable increases in both accuracy and reliability relative to current methods. It is also very applicable in real-life wind power management applications where highly accurate and reliable predictions are required for economic wind power management models and grid stability.

## 6. Conclusions and Future Works

The study has introduced ADPO as a superior version of the Parrot Optimization Algorithm (PO), uniquely designed to deal with the inherent shortcomings of premature convergence and the lack of diversity of the initial formulation. By integrating three radical methods—MDV, DLH, and EAM—ADPO significantly outperforms existing optimization functions, maintaining the same level of algorithm efficiency and wide applicability. Extensive experimental comparison shows that ADPO outperforms various state-of-the-art algorithms on a broad variety of benchmark functions. The assessment ADPO received on the CEC2017 and CEC2022 evaluation suites revealed constant displays of higher ranks of 2.34 and 1.42, respectively. Its excellent performance is not only on theoretical standards but also in practice, as ADPO-LSTM offered the best solutions in wind power prediction at various wind farm stations. ADPO proved incredibly robust and accurate in the spectrum of tasks required in renewable energy forecasting, continuing to show high prediction accuracy, with R^2^ values greater than 0.97 in the extremes of operating conditions and geographical sites. These findings prove that it can be trusted to be a reliable agent in multifaceted modern optimization problems.

The general applicability of this work is significant in the field of optimization, especially for renewable energy applications and the tuning of hyperparameters in neural networks. The fact that ADPO is very similar in performance over a wide selection of problem domains reflects its flexibility as a reliable approach to practical optimization problems. Interestingly, the ability to process hyperparameters in high dimensions fills a crucial niche in modern machine learning tasks, where optimization tasks often deal with a complex parameter surface as well as a high-dimensional time axis.

In spite of all these achievements, ADPO also faces some constraints that should not be overlooked. Sensitivity to population size settings is also observed in highly multimodal search landscapes, where setting an inappropriate population size setting might lead to poor results. Moreover, computational overhead at a very large scale may be significant, particularly in large dimensions, associated with the complicated calculations demanded by the three enhancement mechanisms. In some cases, dimension-wise learning through the DLH strategy can make a momentary jump in the computational complexity, and extra measurements are needed to get the best performance out of the algorithm. These shortcomings suggest the possibility of further improvement.

Multiple directions have been found on which future research will be focused to improve ADPO and increase its applicability. A particularly promising direction is the development of self-adaptive parameter adjustment mechanisms that will increase robustness and reduce the amount of manual configuration. Also, it can be further improved by incorporating machine learning methodology into the intelligent selection of strategies to make it more adaptive and computationally efficient. Moreover, the multi-objective optimization representation of ADPO is vital to solving practical real-life problems with conflicting objectives, especially in complex engineering design scenarios. Furthermore, knowledge- and domain-specific applications will also be improved, including solar power forecasting [69], energy storage optimization [70], renewable energy integration [71], feature selection [72,73], image segmentation [74,75], wireless sensor networks [76,77], task scheduling in cloud computing [78], human activity recognition [79], bioinformatics applications [80], autonomous vehicle path planning [81], software defects [82], medical classification [83,84], gene classification [85], path planning [86], cybersecurity threat detection [87], and dynamic traffic routing [88]. These developments aim to make ADPO a general-purpose optimization system able to solve progressively complex optimization problems whilst retaining its strong foundations of precision, effectiveness, and reliability.

## Figures and Tables

**Table 1:** Various parameter settings.

Algorithm	Parameter Value
IVY	beta1=[1,1.5),GV=[0,1]
HOA	Angle of inclination =[0,50], SF=[1,3]
SWO	TR=0.3, CR=0.2
HHO	$E_{0}$ changes from −1 to 1
AOA	α=5; μ=0.5
KOA	$\bar{T}=3,\;\mu 0=0.1,\;\gamma =15$
GJO	$c_{1}\;varies\;from\;1\;to\;2$
WOA	k=1, q=[−1,1]
CMA-ES	*σ* = 0.5, *μ* = *λ*/2
CSOAOA	μ=0.499, a=5
GWO_CS	a: Linear reduction from 2 to 0
RDWOA	a1=[2,0], a2=[−2,−1], s=0, b=1
jDE	p=0.05, F=0.5 ,c=0.1, CR=0.9
ISSA	PP=0.2, ST=0.8

**Table 2:** Experimental results using CEC2022 for different variants of PO.

F		ADPO	ADPO-DLH	ADPO-EAM	ADPO-MDV	PO
F1	AVG	303.862	1.66 × 10^4^	**300.839**	3185.297	1.83 × 10^4^
	SD	2.899	3853.111	**0.390**	1423.174	4152.061
	RAN	2	4	**1**	3	5
F2	AVG	456.679	525.328	468.321	487.787	501.975
	SD	**12.155**	80.608	37.535	30.722	39.841
	RAN	**1**	5	2	3	4
F3	AVG	**618.668**	650.046	623.121	649.312	657.763
	SD	**4.773**	9.967	12.532	15.584	16.308
	RAN	**1**	4	2	3	5
F4	AVG	**870.459**	893.445	879.201	882.195	887.711
	SD	18.172	18.159	**11.278**	17.248	16.002
	RAN	**1**	5	2	3	4
F5	AVG	**1793.324**	1953.369	2037.207	2395.176	2758.038
	SD	386.598	**165.996**	492.805	392.801	361.537
	RAN	**1**	2	3	4	5
F6	AVG	**4812.973**	9.26 × 10^5^	6862.081	11428.323	2.18 × 10^5^
	SD	**3762.032**	1.39 × 10^6^	6587.417	7408.561	1.75 × 10^5^
	RAN	**1**	5	2	3	4
F7	AVG	**2090.781**	2143.831	2107.010	2128.600	2132.220
	SD	34.803	**27.458**	28.586	30.951	37.998
	RAN	**1**	5	2	3	4
F8	AVG	**2231.654**	2288.420	2240.885	2246.410	2277.449
	SD	**7.976**	61.781	43.257	16.325	57.658
	RAN	**1**	5	2	3	4
F9	AVG	2481.315	2575.709	**2480.978**	2495.260	2594.209
	SD	0.618	36.557	**0.176**	8.134	52.688
	RAN	2	4	**1**	3	5
F10	AVG	**2518.107**	2559.140	2557.694	2758.615	2622.379
	SD	**54.563**	121.507	80.237	813.674	131.213
	RAN	**1**	3	2	5	4
F11	AVG	2951.864	3223.154	**2941.356**	3013.948	3231.580
	SD	51.855	204.779	**50.784**	122.523	111.284
	RAN	2	4	**1**	3	5
F12	AVG	2978.793	3030.089	**2975.518**	2989.338	3040.060
	SD	28.171	68.964	**23.851**	33.773	43.344
	RAN	2	4	**1**	3	5
Average rank		**1.33**	4.17	1.75	3.25	4.50
Final rank		**1**	4	2	3	5
Friedman rank		**1.55**	4.00	1.87	3.24	4.34

**Table 3:** Experimental results using CEC2022 for ADPO and other competitors.

F		ADPO	PO	IVY	HOA	HHO	AOA	SWO	COA	GJO	WOA	KOA
F1	AVG	**301.302**	1.60 × 10^4^	2.40 × 10^4^	2.77 × 10^4^	1.11 × 10^4^	3.38 × 10^4^	2.70 × 10^4^	4.31 × 10^4^	1.65 × 10^4^	2.48 × 10^4^	1.11 × 10^5^
	SD	**0.981**	4.46 × 10^3^	1.01 × 10^4^	6329.500	7244.094	1.17 × 10^4^	7.67 × 10^3^	1.34 × 10^4^	5.42 × 10^3^	9.44 × 10^3^	1.21 × 10^5^
	RAN	**1**	3	5	8	2	9	7	10	4	6	11
F2	AVG	466.259	529.092	**464.089**	1975.235	481.445	2530.853	790.688	2890.766	610.729	570.208	5045.097
	SD	**19.242**	40.681	29.231	465.416	25.270	1023.860	106.168	796.635	78.953	54.887	1488.769
	RAN	2	4	**1**	8	3	9	7	10	6	5	11
F3	AVG	621.667	655.570	**606.720**	663.939	661.088	664.580	650.324	682.391	626.149	674.481	704.271
	SD	10.531	10.121	11.508	9.539	10.085	**7.646**	7.889	7.860	10.040	14.432	10.033
	RAN	2	5	**1**	7	6	8	4	10	3	9	11
F4	AVG	878.170	892.127	**872.859**	931.436	886.788	950.451	962.916	976.083	897.802	932.687	1063.219
	SD	16.148	16.820	19.693	15.451	14.546	15.147	19.739	**14.435**	26.177	31.132	17.626
	RAN	2	4	**1**	6	3	8	9	10	5	7	11
F5	AVG	2013.369	2518.767	2368.061	2749.518	2855.258	2920.390	3476.263	3566.697	**1900.620**	3919.915	9733.317
	SD	402.268	505.119	**182.276**	379.229	268.721	467.652	940.511	355.377	407.825	1104.669	1941.003
	RAN	2	4	3	5	6	7	8	9	**1**	10	11
F6	AVG	**4028.385**	2.45 × 10^5^	1.56 × 10^4^	1.23 × 10^9^	1.37 × 10^5^	8.68 × 10^8^	7.23 × 10^7^	2.59 × 10^9^	9.56 × 10^6^	1.89 × 10^6^	3.43 × 10^9^
	SD	**3172.272**	4.90 × 10^5^	5.95 × 10^4^	7.60 × 10^8^	6.95 × 10^4^	8.86 × 10^8^	6.48 × 10^7^	1.15 × 10^9^	1.12 × 10^7^	6.14 × 10^6^	1.12 × 10^9^
	RAN	**1**	4	2	9	3	8	7	10	6	5	11
F7	AVG	**2103.721**	2143.961	2144.343	2166.174	2205.912	2226.781	2179.929	2222.639	2121.605	2206.460	2356.355
	SD	**29.187**	37.351	75.978	40.021	65.638	93.593	46.392	34.530	47.310	59.227	68.525
	RAN	**1**	3	4	5	7	10	6	9	2	8	11
F8	AVG	2243.629	2297.781	2373.706	2377.147	2255.380	2497.090	2292.650	2432.047	**2240.629**	2274.965	2983.494
	SD	36.205	76.701	153.618	134.631	38.109	182.612	53.938	183.263	**26.135**	65.200	291.237
	RAN	2	6	7	8	3	10	5	9	**1**	4	11
F9	AVG	**2481.000**	2564.784	2483.255	3260.897	2508.538	3091.623	2628.026	3477.854	2584.904	2573.909	3402.602
	SD	**0.282**	35.467	3.124	230.424	22.711	228.864	46.043	353.977	50.466	46.192	238.632
	RAN	**1**	4	2	9	3	8	7	11	6	5	10
F10	AVG	**2539.901**	2778.659	3648.251	5296.652	4088.618	5543.269	3760.872	6381.361	3307.365	4460.976	6499.317
	SD	**103.711**	765.195	1006.841	1352.603	595.663	914.065	1416.084	1214.830	1252.848	1231.506	1325.805
	RAN	**1**	2	4	8	6	9	5	10	3	7	11
F11	AVG	**2935.671**	3310.221	3317.437	7820.076	3003.779	8347.090	5050.069	8742.492	4638.858	3384.729	1.11 × 10^4^
	SD	**138.958**	487.063	1028.409	606.779	139.075	1137.649	623.546	870.316	536.012	270.966	1390.234
	RAN	**1**	3	4	8	2	9	7	10	6	5	11
F12	AVG	**2991.295**	3041.325	3034.367	3846.387	3185.708	3807.392	3261.218	3646.558	3027.704	3086.782	3854.341
	SD	51.654	**41.210**	91.186	210.462	137.706	236.661	71.964	231.355	57.378	137.904	189.245
	RAN	**1**	4	3	10	6	9	7	8	2	5	11
Average rank		**1.42**	3.83	3.08	7.58	4.17	8.67	6.58	9.67	3.75	6.33	10.92
Final rank		**1**	4	2	8	5	9	7	10	3	6	11

**Table 4:** The Wilcoxon *p*-values of ADPO versus other algorithms using CEC2022.

F	ADPO	PO	IVY	HOA	HHO	AOA	SWO	COA	GJO	WOA	KOA
F1	1.50990 × 10^−11^	1.50990 × 10^−11^	1.50990 × 10^−11^	1.50990 × 10^−11^	1.50990 × 10^−11^	1.50990 × 10^−11^	1.50990 × 10^−11^	1.50990 × 10^−11^	1.50990 × 10^−11^	1.50990 × 10^−11^	1.50990 × 10^−11^
F2	2.48760 × 10^−11^	1.50990 × 10^−11^	6.79720 × 10^−8^	1.84490 × 10^−11^	3.03290 × 10^−11^	2.73100 × 10^−6^	1.50990 × 10^−11^	8.03110 × 10^−7^	4.24240 × 10^−9^	9.28370 × 10^−10^	2.48760 × 10^−11^
F3	1.50990 × 10^−11^	1.50990 × 10^−11^	1.50990 × 10^−11^	4.15730 × 10^−3^	1.50990 × 10^−11^	7.39400 × 10^−1^	1.50990 × 10^−11^	1.67600 × 10^−8^	1.50990 × 10^−11^	1.11360 × 10^−9^	1.50990 × 10^−11^
F4	1.50990 × 10^−11^	1.50990 × 10^−11^	2.78060 × 10^−4^	2.28630 × 10^−9^	1.50990 × 10^−11^	2.01650 × 10^−3^	1.50990 × 10^−11^	7.64580 × 10^−6^	2.03860 × 10^−11^	1.68410 × 10^−5^	1.50990 × 10^−11^
F5	1.50990 × 10^−11^	1.50990 × 10^−11^	2.74700 × 10^−11^	2.35690 × 10^−4^	1.50990 × 10^−11^	1.02330 × 10^−1^	1.50990 × 10^−11^	2.09130 × 10^−9^	1.50990 × 10^−11^	1.07720 × 10^−10^	1.50990 × 10^−11^
F6	1.66920 × 10^−11^	1.50990 × 10^−11^	1.25970 × 10^−1^	2.74700 × 10^−11^	2.25220 × 10^−11^	1.00000 × 10^+00^	1.50990 × 10^−11^	5.46830 × 10^−11^	1.07720 × 10^−10^	2.03860 × 10^−11^	1.66920 × 10^−11^
F7	1.50990 × 10^−11^	1.50990 × 10^−11^	4.94170 × 10^−3^	1.46030 × 10^−2^	2.25220 × 10^−11^	2.17020 × 10^−1^	1.50990 × 10^−11^	4.15730 × 10^−3^	1.50990 × 10^−11^	3.06050 × 10^−10^	1.50990 × 10^−11^
F8	1.46080 × 10^−9^	1.50990 × 10^−11^	2.70710 × 10^−1^	6.43520 × 10^−10^	2.54610 × 10^−8^	2.78060 × 10^−4^	1.50990 × 10^−11^	8.40660 × 10^−5^	6.01160 × 10^−9^	1.84490 × 10^−11^	1.46080 × 10^−9^
F9	1.50990 × 10^−11^	1.50990 × 10^−11^	1.66920 × 10^−11^	1.50990 × 10^−11^	1.50990 × 10^−11^	8.64990 × 10^−1^	1.50990 × 10^−11^	4.49670 × 10^−11^	1.50990 × 10^−11^	1.50990 × 10^−11^	1.50990 × 10^−11^
F10	5.86870 × 10^−10^	1.66920 × 10^−11^	2.70710 × 10^−1^	4.87780 × 10^−10^	6.55550 × 10^−9^	5.15730 × 10^−3^	1.50990 × 10^−11^	2.31950 × 10^−5^	7.05490 × 10^−10^	1.43580 × 10^−10^	5.86870 × 10^−10^
F11	6.43520 × 10^−10^	5.86870 × 10^−10^	7.97820 × 10^−2^	8.39880 × 10^−4^	4.42050 × 10^−7^	5.57130 × 10^−4^	1.50990 × 10^−11^	4.14600 × 10^−6^	6.79720 × 10^−8^	1.38630 × 10^−5^	6.43520 × 10^−10^
F12	1.50990 × 10^−11^	1.50990 × 10^−11^	1.50990 × 10^−11^	1.50990 × 10^−11^	1.50990 × 10^−11^	1.09790 × 10^−7^	1.50990 × 10^−11^	1.50990 × 10^−11^	1.50990 × 10^−11^	1.50990 × 10^−11^	1.50990 × 10^−11^

**Table 5:** Experimental results using CEC2017 for ADPO and advanced competitors, 50D.

F		ADPO	eCOA	CMAES	DAOA	CSOAOA	GWO_CS	RDWOA	jDE	ISSA	IPSO_IGSA
F1	AVG	**2.43 × 10^6^**	8.08 × 10^9^	1.95 × 10^10^	2.62 × 10^11^	2.09 × 10^8^	1.42 × 10^10^	2.11 × 10^10^	1.23 × 10^11^	2.27 × 10^8^	4.11 × 10^10^
	SD	**2.16 × 10^6^**	1.24 × 10^9^	3.03 × 10^10^	1.46 × 10^10^	7.33 × 10^7^	4.30 × 10^9^	2.38 × 10^9^	7.75 × 10^10^	6.78 × 10^7^	1.38 × 10^10^
	RAN	**1**	4	6	10	2	5	7	9	3	8
F3	AVG	**2.79 × 10^4^**	1.29 × 10^5^	3.82 × 10^5^	5.74 × 10^7^	1.17 × 10^5^	1.23 × 10^5^	1.89 × 10^5^	2.88 × 10^5^	2.47 × 10^5^	1.70 × 10^5^
	SD	**8260.825**	1.21 × 10^4^	4.93 × 10^4^	1.35 × 10^8^	1.18 × 10^4^	1.77 × 10^4^	1.45 × 10^4^	4.19 × 10^4^	8.13 × 10^4^	1.94 × 10^4^
	RAN	**1**	4	9	10	2	3	6	8	7	5
F4	AVG	**616.358**	1465.648	8332.552	1.30 × 10^5^	669.436	1496.651	4063.559	5.20 × 10^4^	718.095	8698.568
	SD	48.544	443.718	3064.805	2.80 × 10^4^	41.225	210.031	887.200	8380.828	**31.919**	1866.557
	RAN	**1**	4	7	10	2	5	6	9	3	8
F5	AVG	840.354	842.542	**549.891**	1697.942	828.289	809.189	1043.451	1403.854	881.798	855.080
	SD	63.807	54.380	**3.361**	91.762	34.561	23.177	28.878	43.358	26.887	106.625
	RAN	4	5	**1**	10	3	2	8	9	7	6
F6	AVG	650.472	660.316	**621.711**	757.645	630.582	632.558	682.780	701.936	667.615	671.656
	SD	7.193	9.409	33.627	8.127	15.080	7.842	8.880	22.860	**2.638**	19.663
	RAN	4	5	**1**	10	2	3	8	9	6	7
F7	AVG	1342.796	1421.933	**956.599**	6057.349	1451.628	1180.698	1764.588	3617.830	1673.438	1801.288
	SD	142.095	192.150	155.021	466.162	162.112	**96.478**	112.411	1358.797	146.308	103.252
	RAN	3	4	**1**	10	5	2	7	9	6	8
F8	AVG	1161.837	1110.964	1178.717	2017.948	1158.436	**1108.937**	1321.955	1650.974	1173.708	1169.514
	SD	61.530	**39.309**	257.356	73.263	40.599	41.371	58.026	200.700	46.786	49.201
	RAN	4	2	7	10	3	**1**	8	9	6	5
F9	AVG	1.44 × 10^4^	1.30 × 10^4^	**900.151**	1.17 × 10^5^	1.15 × 10^4^	1.51 × 10^4^	2.72 × 10^4^	6.14 × 10^4^	1.56 × 10^4^	1.57 × 10^4^
	SD	1699.155	563.595	**0.235**	1.50 × 10^4^	1019.419	8516.092	2781.981	8150.298	1433.657	6902.928
	RAN	4	3	**1**	10	2	5	8	9	6	7
F10	AVG	7718.304	8311.301	1.50 × 10^4^	1.69 × 10^4^	**6484.701**	7483.089	1.26 × 10^4^	1.54 × 10^4^	8521.760	1.49 × 10^4^
	SD	724.850	762.366	**348.879**	588.528	921.221	1289.202	1004.835	566.957	1119.243	845.519
	RAN	3	4	8	10	**1**	2	6	9	5	7
F11	AVG	**1489.778**	2157.984	6.14 × 10^4^	1.14 × 10^5^	3037.103	7629.620	5496.289	3.83 × 10^4^	2492.502	1.74 × 10^4^
	SD	**83.804**	322.622	2.07 × 10^4^	8.49 × 10^4^	326.477	2509.494	1162.624	4553.782	283.031	3825.336
	RAN	**1**	2	9	10	4	6	5	8	3	7
F12	AVG	3.26 × 10^7^	2.31 × 10^8^	2.42 × 10^10^	1.60 × 10^11^	**2.66 × 10^7^**	1.56 × 10^9^	4.25 × 10^9^	6.21 × 10^10^	4.84 × 10^7^	7.34 × 10^9^
	SD	**2.00 × 10^7^**	1.07 × 10^8^	4.70 × 10^9^	1.58 × 10^10^	2.60 × 10^7^	6.91 × 10^8^	3.93 × 10^9^	1.24 × 10^10^	3.26 × 10^7^	2.56 × 10^9^
	RAN	2	4	8	10	**1**	5	6	9	3	7
F13	AVG	**4.67 × 10^4^**	1.92 × 10^5^	1.14 × 10^10^	9.09 × 10^10^	4.98 × 10^5^	8.85 × 10^7^	4.06 × 10^8^	2.71 × 10^10^	6.71 × 10^4^	3.10 × 10^8^
	SD	**7.32 × 10^3^**	3.57 × 10^5^	2.70 × 10^9^	1.86 × 10^10^	3.71 × 10^5^	3.16 × 10^7^	2.23 × 10^8^	1.64 × 10^10^	2.44 × 10^4^	4.35 × 10^8^
	RAN	**1**	3	8	10	4	5	7	9	2	6
F14	AVG	**2.59 × 10^5^**	9.18 × 10^5^	2.12 × 10^7^	3.26 × 10^8^	1.67 × 10^6^	1.38 × 10^6^	6.76 × 10^6^	2.27 × 10^7^	1.48 × 10^6^	8.51 × 10^6^
	SD	**1.33 × 10^5^**	5.70 × 10^5^	1.33 × 10^7^	2.19 × 10^8^	1.10 × 10^6^	1.08 × 10^6^	5.70 × 10^6^	7.78 × 10^6^	6.99 × 10^5^	8.61 × 10^6^
	RAN	**1**	2	8	10	5	3	6	9	4	7
F15	AVG	**2.08 × 10^4^**	2.81 × 10^4^	1.38 × 10^9^	3.28 × 10^10^	5.33 × 10^4^	2.55 × 10^7^	7.85 × 10^7^	7.45 × 10^9^	2.44 × 10^4^	5.55 × 10^7^
	SD	**6.09 × 10^3^**	7.25 × 10^3^	4.23 × 10^8^	1.28 × 10^10^	2.96 × 10^4^	3.76 × 10^7^	7.93 × 10^7^	5.03 × 10^9^	1.18 × 10^4^	1.15 × 10^8^
	RAN	**1**	3	8	10	4	5	7	9	2	6
F16	AVG	4029.422	3861.616	6539.304	1.65 × 10^4^	**3239.377**	3395.411	5943.220	8258.053	4081.894	4265.301
	SD	367.002	**322.451**	523.678	3021.001	669.226	322.737	618.996	1418.040	632.085	533.700
	RAN	4	3	8	10	**1**	2	7	9	5	6
F17	AVG	3260.336	3634.634	**2665.323**	520,653.499	3297.556	3160.881	4222.925	19,889.934	3616.944	3787.720
	SD	537.823	369.929	**250.825**	422,719.420	260.826	281.760	468.911	17,601.719	429.145	532.911
	RAN	3	6	**1**	10	4	2	8	9	5	7
F18	AVG	3.48 × 10^6^	**2.75 × 10^6^**	1.13 × 10^8^	4.64 × 10^8^	2.89 × 10^6^	9.20 × 10^6^	3.00 × 10^7^	1.32 × 10^8^	5.26 × 10^6^	1.16 × 10^7^
	SD	2.16 × 10^6^	1.20 × 10^6^	2.49 × 10^7^	1.41 × 10^8^	**8.35 × 10^5^**	7.96 × 10^6^	2.46 × 10^7^	4.24 × 10^7^	5.84 × 10^6^	8.37 × 10^6^
	RAN	3	**1**	8	10	2	5	7	9	4	6
F19	AVG	4.95 × 10^4^	3.96 × 10^5^	1.31 × 10^9^	1.63 × 10^10^	**2.67 × 10^4^**	6.33 × 10^6^	9.78 × 10^6^	2.50 × 10^9^	3.73 × 10^4^	5.50 × 10^5^
	SD	1.96 × 10^4^	4.75 × 10^4^	7.71 × 10^8^	3.63 × 10^9^	**5.97 × 10^3^**	1.14 × 10^7^	6.49 × 10^6^	1.17 × 10^9^	1.24 × 10^4^	3.68 × 10^5^
	RAN	3	4	8	10	**1**	6	7	9	2	5
F20	AVG	**3066.824**	3223.354	3755.252	5229.949	3169.382	3079.606	3439.724	4310.467	3547.154	3871.279
	SD	222.742	312.746	244.158	279.803	428.861	130.822	**108.685**	353.531	213.676	214.596
	RAN	**1**	4	7	10	3	2	5	9	6	8
F21	AVG	2662.173	2636.420	2625.002	3638.206	2650.265	**2538.095**	2978.520	3212.749	2844.101	2731.186
	SD	79.079	36.434	299.277	119.440	42.254	**26.879**	102.297	122.748	34.360	87.016
	RAN	5	3	2	10	4	**1**	8	9	7	6
F22	AVG	**7956.579**	1.12 × 10^4^	1.68 × 10^4^	1.85 × 10^4^	8714.231	8372.384	1.35 × 10^4^	1.71 × 10^4^	1.07 × 10^4^	1.64 × 10^4^
	SD	4437.808	842.920	**189.802**	1001.036	3166.453	2254.177	736.221	373.442	490.879	399.462
	RAN	**1**	5	8	10	3	2	6	9	4	7
F23	AVG	3198.328	3174.853	3413.438	5257.213	**3043.978**	3075.586	3639.131	4205.040	3482.904	3874.196
	SD	104.785	65.958	48.089	401.928	**46.192**	52.803	63.119	160.957	138.772	219.283
	RAN	4	3	5	10	**1**	2	7	9	6	8
F24	AVG	3446.034	3266.602	3496.998	5962.147	3516.754	**3246.786**	3716.717	4521.426	3596.922	4138.812
	SD	140.581	83.274	**54.391**	533.209	115.301	89.314	91.453	296.736	119.341	135.882
	RAN	3	2	4	10	5	**1**	7	9	6	8
F25	AVG	**3095.805**	3728.568	4054.579	5.84 × 10^4^	3193.396	4070.799	5167.651	3.04 × 10^4^	3212.249	7009.422
	SD	28.311	192.851	1625.322	5190.684	**11.453**	435.175	404.137	3538.472	46.614	1396.288
	RAN	**1**	4	5	10	2	6	7	9	3	8
F26	AVG	**5827.614**	1.16 × 10^4^	1.12 × 10^4^	3.01 × 10^4^	8143.500	6213.015	1.32 × 10^4^	2.13 × 10^4^	1.05 × 10^4^	1.39 × 10^4^
	SD	3845.466	924.356	**70.470**	3428.947	3425.603	1555.076	785.538	1039.129	3431.660	1758.167
	RAN	**1**	6	5	10	3	2	7	9	4	8
F27	AVG	3626.228	3866.233	3903.322	8778.909	**3606.561**	3687.598	4641.606	6086.369	3844.967	5882.212
	SD	228.514	133.222	55.881	744.725	172.600	**41.701**	421.257	890.516	162.791	645.253
	RAN	2	5	6	10	**1**	3	7	9	4	8
F28	AVG	**3357.193**	4486.564	1.00 × 10^4^	2.49 × 10^4^	3493.394	4404.121	5969.609	1.58 × 10^4^	3574.933	6795.098
	SD	**32.968**	217.558	440.044	4179.754	57.593	303.131	330.416	1476.058	118.711	401.804
	RAN	**1**	5	8	10	2	4	6	9	3	7
F29	AVG	4581.788	6753.843	1.33 × 10^4^	2.58 × 10^6^	**4520.731**	4931.909	8368.144	1.94 × 10^4^	5576.914	9624.458
	SD	367.149	538.216	4948.482	1.77 × 10^6^	**251.205**	422.919	665.707	6958.151	493.070	2865.338
	RAN	2	5	8	10	**1**	3	6	9	4	7
F30	AVG	9.03 × 10^6^	1.47 × 10^8^	2.12 × 10^9^	2.43 × 10^10^	**1.04 × 10^6^**	1.76 × 10^8^	3.01 × 10^8^	2.92 × 10^9^	3.76 × 10^6^	2.15 × 10^8^
	SD	4.25 × 10^6^	1.69 × 10^7^	1.01 × 10^9^	4.30 × 10^9^	**1.64 × 10^5^**	6.86 × 10^7^	8.12 × 10^7^	1.90 × 10^9^	6.31 × 10^5^	3.65 × 10^7^
	RAN	3	4	8	10	**1**	5	7	9	2	6
Average rank		**2.34**	3.76	5.97	10.00	2.55	3.38	6.79	8.93	4.41	6.86
Final rank		**1**	4	6	10	2	3	7	9	5	8

**Table 6:** The Wilcoxon *p*-values for ADPO versus other advanced algorithms using CEC2017.

F	ADPO	eCOA	CMAES	DAOA	CSOAOA	GWO_CS	RDWOA	jDE	ISSA	IPSO_IGSA
F1	3.93940 × 10^−1^	1.08230 × 10^−11^	1.08230 × 10^−11^	1.08230 × 10^−11^	1.08230 × 10^−11^	1.08230 × 10^−11^	1.08230 × 10^−11^	1.08230 × 10^−11^	1.08230 × 10^−11^	3.93940 × 10^−1^
F3	1.08230 × 10^−11^	9.92420 × 10^−1^	1.08230 × 10^−11^	6.99130 × 10^−1^	1.08230 × 10^−11^	3.09520 × 10^−1^	1.08230 × 10^−11^	2.40260 × 10^−1^	1.08230 × 10^−11^	1.08230 × 10^−11^
F4	1.08230 × 10^−11^	1.08230 × 10^−11^	1.08230 × 10^−11^	1.08230 × 10^−11^	1.08230 × 10^−11^	1.08230 × 10^−11^	1.08230 × 10^−11^	1.08230 × 10^−11^	1.08230 × 10^−11^	1.08230 × 10^−11^
F5	1.08230 × 10^−11^	4.84850 × 10^−1^	1.08230 × 10^−11^	1.08230 × 10^−11^	1.08230 × 10^−11^	1.08230 × 10^−11^	1.08230 × 10^−11^	1.08230 × 10^−11^	1.08230 × 10^−11^	1.08230 × 10^−11^
F6	1.08230 × 10^−11^	1.08230 × 10^−11^	1.08230 × 10^−11^	1.08230 × 10^−11^	1.08230 × 10^−11^	1.32030 × 10^−1^	1.08230 × 10^−11^	1.00000 × 10^+00^	1.08230 × 10^−11^	1.08230 × 10^−11^
F7	1.08230 × 10^−11^	1.08230 × 10^−11^	1.08230 × 10^−11^	2.40260 × 10^−1^	1.08230 × 10^−11^	1.08230 × 10^−11^	1.08230 × 10^−11^	1.08230 × 10^−11^	1.08230 × 10^−11^	1.08230 × 10^−11^
F8	1.08230 × 10^−11^	1.29870 × 10^−9^	1.08230 × 10^−11^	1.08230 × 10^−11^	3.09520 × 10^−1^	1.08230 × 10^−11^	1.08230 × 10^−11^	1.08230 × 10^−11^	1.08230 × 10^−11^	1.08230 × 10^−11^
F9	1.08230 × 10^−11^	9.87010 × 10^−1^	1.08230 × 10^−11^	9.95670 × 10^−1^	5.88740 × 10^−1^	5.88740 × 10^−1^	1.08230 × 10^−11^	1.08230 × 10^−11^	1.08230 × 10^−11^	1.08230 × 10^−11^
F10	9.30740 × 10^−9^	6.99130 × 10^−1^	1.08230 × 10^−11^	6.99130 × 10^−1^	1.32030 × 10^−1^	1.08230 × 10^−11^	7.57580 × 10^−11^	1.32030 × 10^−1^	1.08230 × 10^−11^	9.30740 × 10^−9^
F11	1.08230 × 10^−11^	5.88740 × 10^−1^	1.08230 × 10^−11^	4.84850 × 10^−1^	2.05630 × 10^−9^	1.00000 × 10^+00^	1.29870 × 10^−9^	4.84850 × 10^−1^	1.08230 × 10^−11^	1.08230 × 10^−11^
F12	1.08230 × 10^−11^	9.95670 × 10^−1^	1.08230 × 10^−11^	1.29870 × 10^−9^	1.08230 × 10^−11^	2.40260 × 10^−1^	1.08230 × 10^−11^	1.08230 × 10^−11^	1.08230 × 10^−11^	1.08230 × 10^−11^
F13	1.08230 × 10^−11^	9.37230 × 10^−1^	1.08230 × 10^−11^	9.37230 × 10^−1^	1.08230 × 10^−11^	1.08230 × 10^−11^	2.16450 × 10^−11^	1.08230 × 10^−11^	1.08230 × 10^−11^	1.08230 × 10^−11^
F14	1.00000 × 10^+00^	1.08230 × 10^−11^	1.08230 × 10^−11^	1.00000 × 10^+00^	2.40260 × 10^−01^	1.08230 × 10^−11^	1.08230 × 10^−11^	1.08230 × 10^−11^	1.08230 × 10^−11^	1.00000 × 10^+00^
F15	1.08230 × 10^−11^	1.00000 × 10^+00^	1.08230 × 10^−11^	6.99130 × 10^−01^	1.08230 × 10^−11^	6.99130 × 10^−01^	1.08230 × 10^−11^	1.08230 × 10^−11^	1.08230 × 10^−11^	1.08230 × 10^−11^
F16	2.16450 × 10^−11^	9.97840 × 10^−1^	1.08230 × 10^−11^	1.08230 × 10^−11^	1.08230 × 10^−11^	2.16450 × 10^−11^	1.08230 × 10^−11^	1.08230 × 10^−11^	1.08230 × 10^−11^	2.16450 × 10^−11^
F17	5.88740 × 10^−1^	1.08230 × 10^−11^	1.08230 × 10^−11^	9.92420 × 10^−1^	1.08230 × 10^−11^	1.08230 × 10^−11^	2.16450 × 10^−11^	9.87010 × 10^−1^	1.08230 × 10^−11^	5.88740 × 10^−1^
F18	1.08230 × 10^−11^	1.08230 × 10^−11^	1.08230 × 10^−11^	1.08230 × 10^−11^	1.08230 × 10^−11^	2.16450 × 10^−11^	1.08230 × 10^−11^	1.08230 × 10^−11^	1.08230 × 10^−11^	1.08230 × 10^−11^
F19	2.16450 × 10^−11^	1.08230 × 10^−11^	1.08230 × 10^−11^	1.08230 × 10^−11^	1.08230 × 10^−11^	2.05630 × 10^−9^	1.08230 × 10^−11^	4.32900 × 10^−11^	1.08230 × 10^−11^	2.16450 × 10^−11^
F20	6.49350 × 10^−9^	8.18180 × 10^−1^	1.08230 × 10^−11^	3.93940 × 10^−1^	1.08230 × 10^−11^	9.30740 × 10^−9^	1.08230 × 10^−11^	1.08230 × 10^−11^	1.08230 × 10^−11^	6.49350 × 10^−9^
F21	1.08230 × 10^−11^	1.08230 × 10^−11^	1.08230 × 10^−11^	1.08230 × 10^−11^	1.08230 × 10^−11^	1.08230 × 10^−11^	1.08230 × 10^−11^	1.08230 × 10^−11^	1.08230 × 10^−11^	1.08230 × 10^−11^
F22	1.08230 × 10^−11^	5.88740 × 10^−1^	1.08230 × 10^−11^	1.08230 × 10^−11^	1.08230 × 10^−11^	1.08230 × 10^−11^	1.08230 × 10^−11^	1.08230 × 10^−11^	1.08230 × 10^−11^	1.08230 × 10^−11^
F23	1.08230 × 10^−11^	1.08230 × 10^−11^	1.08230 × 10^−11^	1.08230 × 10^−11^	1.08230 × 10^−11^	1.08230 × 10^−11^	1.08230 × 10^−11^	1.08230 × 10^−11^	1.08230 × 10^−11^	1.08230 × 10^−11^
F24	1.08230 × 10^−11^	1.00000 × 10^+00^	1.08230 × 10^−11^	1.08230 × 10^−11^	1.08230 × 10^−11^	9.95670 × 10^−1^	1.08230 × 10^−11^	1.08230 × 10^−11^	1.08230 × 10^−11^	1.08230 × 10^−11^
F25	1.08230 × 10^−11^	1.08230 × 10^−11^	1.08230 × 10^−11^	1.08230 × 10^−11^	1.08230 × 10^−11^	1.08230 × 10^−11^	1.08230 × 10^−11^	1.08230 × 10^−11^	1.08230 × 10^−11^	1.08230 × 10^−11^
F26	1.00000 × 10^+00^	1.08230 × 10^−11^	1.08230 × 10^−11^	1.08230 × 10^−11^	1.00000 × 10^+00^	1.79650 × 10^−1^	1.08230 × 10^−11^	1.00000 × 10^+00^	1.08230 × 10^−11^	1.00000 × 10^+00^
F27	3.93940 × 10^−1^	9.95670 × 10^−1^	1.08230 × 10^−11^	1.08230 × 10^−11^	9.30740 × 10^−9^	1.08230 × 10^−11^	1.08230 × 10^−11^	1.08230 × 10^−11^	1.08230 × 10^−11^	3.93940 × 10^−1^
F28	1.00000 × 10^+00^	1.08230 × 10^−11^	1.08230 × 10^−11^	1.08230 × 10^−11^	1.08230 × 10^−11^	4.32900 × 10^−11^	1.08230 × 10^−11^	1.08230 × 10^−11^	1.08230 × 10^−11^	1.00000 × 10^+00^
F29	3.93940 × 10^−1^	1.08230 × 10^−11^	1.08230 × 10^−11^	1.08230 × 10^−11^	1.00000 × 10^+00^	1.08230 × 10^−11^	1.08230 × 10^−11^	1.08230 × 10^−11^	1.08230 × 10^−11^	3.93940 × 10^−1^
F30	1.00000 × 10^+00^	1.08230 × 10^−11^	1.08230 × 10^−11^	1.08230 × 10^−11^	9.37230 × 10^−1^	1.08230 × 10^−11^	1.08230 × 10^−11^	6.49350 × 10^−9^	1.08230 × 10^−11^	1.00000 × 10^+00^

**Table 7:** Experimental average running results using CEC2022.

F	ADPO	PO	IVY	HOA	HHO	AOA	SWO	COA	GJO	WOA	KOA
F1	0.90527	0.30711	1.02636	0.13643	0.24529	0.19685	0.00808	0.19791	0.37429	0.10800	0.01245
F2	0.83592	0.33225	0.85256	0.14001	0.24348	0.18791	0.00458	0.18577	0.41604	0.09141	0.00826
F3	1.11334	0.35110	0.83730	0.25103	0.58962	0.27909	0.00830	0.41503	0.42706	0.18210	0.01042
F4	0.95505	0.26727	0.73182	0.15731	0.30116	0.20894	0.00541	0.24958	0.35999	0.11791	0.00826
F5	1.02721	0.28030	0.75854	0.18023	0.37381	0.22601	0.00623	0.27336	0.38006	0.12129	0.00790
F6	0.79794	0.25164	0.85514	0.16841	0.26711	0.20225	0.00522	0.21654	0.34851	0.10210	0.00808
F7	1.31417	0.39658	0.99009	0.34585	0.58004	0.34228	0.01075	0.62644	0.49630	0.24503	0.01185
F8	1.40221	0.53759	1.04832	0.35390	0.60387	0.34604	0.01059	0.60033	0.46907	0.25624	0.01484
F9	1.13334	0.35283	0.82745	0.25306	0.48302	0.30826	0.00853	0.49496	0.43338	0.21182	0.01091
F10	0.99577	0.30504	0.72540	0.20768	0.40904	0.25597	0.00769	0.38480	0.39198	0.17093	0.00990
F11	1.27403	0.39561	0.86463	0.29485	0.57875	0.34540	0.00983	0.58785	0.47009	0.25866	0.01223
F12	1.35035	0.42902	0.87337	0.31900	0.63377	0.37125	0.01099	0.65977	0.50257	0.28452	0.01371

**Table 8:** Experimental average running results using CEC2017 50D.

F	ADPO	eCOA	CMAES	DAOA	CSOAOA	GWO_CS	RDWOA	jDE	ISSA	IPSO_IGSA
F1	1.67386	1.04293	4.24935	0.38412	1.11464	1.95731	0.74037	1.93376	0.50250	0.70356
F3	1.24174	0.78274	3.18205	0.34024	0.68268	1.48834	0.57144	1.59181	0.40555	0.60406
F4	1.27119	0.79545	3.65005	0.36421	0.71415	1.68225	0.61663	1.74907	0.54113	0.69053
F5	1.26769	0.74569	3.25317	0.35912	0.69168	1.48907	0.55943	1.71071	0.43242	0.63177
F6	1.24742	0.76707	4.10569	0.35237	0.70382	1.61792	0.58115	1.63057	0.53805	0.70901
F7	1.91488	1.35427	3.60396	0.64330	1.53468	2.08135	1.59306	2.45592	1.03826	1.03672
F8	1.52636	0.81190	3.90357	0.51789	0.88587	1.77702	0.76409	1.98049	0.65625	0.79616
F9	1.66943	0.80809	3.76615	0.38082	0.76675	1.63850	0.63280	2.10379	0.56871	0.70744
F10	1.37915	0.79232	3.59767	0.38425	0.80669	1.62632	0.66185	1.67851	0.49674	0.65528
F11	1.79548	1.28484	3.87048	0.53070	1.27787	1.95214	1.29371	2.11562	0.80681	0.85990
F12	1.37442	0.93949	4.00364	0.39737	0.80870	1.85520	0.81436	2.94907	0.60320	0.78276
F13	1.71143	1.04850	3.78872	0.49357	1.00667	1.62320	0.75793	1.99845	0.58631	0.73249
F14	1.43323	0.78878	3.40117	0.42298	0.85382	1.74562	0.73677	1.92145	0.55565	0.77742
F15	1.63211	1.06965	4.00738	0.46520	0.94881	1.72975	0.90415	1.93334	0.72383	0.74677
F16	1.28576	0.74910	3.45059	0.39278	0.79488	1.67217	0.71246	1.75685	0.46508	0.66763
F17	1.45320	0.86406	3.79483	0.39798	0.83967	1.56689	0.78986	1.76792	0.55344	0.70001
F18	1.46303	0.96405	3.25036	0.44445	1.02542	1.61823	0.99470	1.87846	0.68746	0.72306
F19	1.33661	0.82929	3.44134	0.40965	0.88940	1.74197	0.65882	1.78277	0.50382	0.66813
F20	2.66244	1.95475	3.91604	0.68843	1.59952	2.00164	1.88637	2.53899	1.29412	0.93342
F21	1.56125	1.15591	3.84839	0.51652	1.22942	1.78423	1.24303	2.07499	0.86073	0.87118
F22	2.76479	2.04891	4.02151	0.77052	2.01565	2.08038	1.97774	2.48590	1.23581	0.94357
F23	2.58576	2.10752	3.85183	0.85031	2.25358	2.09832	2.40603	2.28780	1.40034	1.04877
F24	2.72834	2.28436	4.08418	0.92072	2.32758	1.98287	2.42022	2.46055	1.76056	1.10898
F25	2.67659	2.41989	3.69647	0.81594	2.20327	2.03049	2.32264	3.23136	1.73430	1.03920
F26	2.67151	2.26036	3.73109	0.93509	2.26509	2.01687	2.91336	2.86992	1.91023	1.24187
F27	3.90093	2.87358	5.38594	1.29246	3.48890	2.72201	4.13461	3.70081	2.99426	2.10145
F28	5.33060	4.42535	6.08019	1.73411	4.00612	3.18132	4.94835	3.89686	3.51751	2.25040
F29	4.84923	3.79245	5.73153	1.50264	4.07170	3.53292	4.11101	3.95262	3.62204	2.02870
F30	4.16924	3.24062	6.04186	1.11887	2.95649	2.62767	3.38150	3.58916	2.24090	1.72986

**Table 9:** Parameter ranges for ADPO-LSTM.

Algorithm	Parameter Value	Parameter Range
Number of Hidden Units	$N_{h}$	[20, 150]
Maximum Epochs	$M_{E}$	[20, 200]
Optimization Method	$O_{M}$	1, 2, 3 for SGDM (1), Adam (2), or RMSProp (3)
Minimum Batch Size	$M_{B}$	[64, 256]
Learning Rate Drop Factor	$L_{F}$	[0.1, 0.9]

**Table 10:** Training results over Stations A and B.

	Station A				Station B			
	R^2^	RMSE	MAE	COV	R^2^	RMSE	MAE	COV
LSTM	0.6875	0.0024	0.0017	85.2153	0.6385	0.0026	0.0022	115.8742
PO-LSTM	0.8485	0.0014	0.0007	51.2458	0.8475	0.0012	0.0015	60.5874
SCA-LSTM	0.8425	0.0015	0.0009	53.7894	0.8365	0.0014	0.0016	63.2145
WOA-LSTM	0.8285	0.0017	0.0011	59.8547	0.8245	0.0016	0.0018	66.9874
SOA-LSTM	0.8385	0.0015	0.0010	55.7412	0.8325	0.0015	0.0017	64.5789
HHO-LSTM	0.8515	0.0013	0.0008	50.9685	0.8495	0.0013	0.0014	59.8524
ADPO-LSTM	0.9875	0.0002	0.0001	15.8745	0.9851	0.0004	0.0002	23.7412

**Table 11:** Training results over Stations C and D.

	Station C				Station D			
	R^2^	RMSE	MAE	COV	R^2^	RMSE	MAE	COV
LSTM	0.6185	0.0025	0.0020	101.5874	0.6325	0.0030	0.0022	96.8745
PO-LSTM	0.8125	0.0017	0.0012	63.8745	0.8085	0.0013	0.0012	62.7854
SCA-LSTM	0.8065	0.0018	0.0013	66.2145	0.8075	0.0015	0.0013	65.1478
WOA-LSTM	0.7945	0.0020	0.0015	69.8745	0.7975	0.0017	0.0014	68.3654
SOA-LSTM	0.8025	0.0018	0.0014	67.5896	0.8045	0.0016	0.0013	66.9874
HHO-LSTM	0.8145	0.0016	0.0011	62.1478	0.8125	0.0014	0.0011	61.5478
ADPO-LSTM	0.9758	0.0002	0.0001	25.4578	0.9685	0.0005	0.0003	31.2874

**Table 12:** Testing results over Stations A and B.

	Station A				Station B			
	R^2^	RMSE	MAE	COV	R^2^	RMSE	MAE	COV
LSTM	0.6625	0.0034	0.0026	88.7458	0.5785	0.0037	0.0034	117.8965
PO-LSTM	0.8485	0.0026	0.0019	53.7851	0.8105	0.0020	0.0016	63.4785
SCA-LSTM	0.8165	0.0027	0.0020	56.8547	0.7945	0.0022	0.0018	65.9874
WOA-LSTM	0.7945	0.0029	0.0022	61.2458	0.7615	0.0025	0.0020	70.3654
SOA-LSTM	0.8025	0.0028	0.0021	58.9874	0.7845	0.0023	0.0019	67.8745
HHO-LSTM	0.8465	0.0025	0.0018	52.9635	0.8125	0.0019	0.0015	62.7854
ADPO-LSTM	0.9785	0.0009	0.0008	21.5874	0.9798	0.0010	0.0005	27.4578

**Table 13:** Testing results over Stations C and D.

	Station C				Station D			
	R^2^	RMSE	MAE	COV	R^2^	RMSE	MAE	COV
LSTM	0.5985	0.0037	0.0032	107.5896	0.6105	0.0039	0.0030	102.8745
PO-LSTM	0.7705	0.0021	0.0017	68.1478	0.7695	0.0019	0.0016	68.7854
SCA-LSTM	0.7345	0.0023	0.0019	71.2587	0.7385	0.0021	0.0017	70.1478
WOA-LSTM	0.7085	0.0026	0.0021	74.8745	0.7215	0.0024	0.0019	73.5896
SOA-LSTM	0.7245	0.0024	0.0020	72.9874	0.7325	0.0022	0.0018	71.8745
HHO-LSTM	0.7725	0.0020	0.0016	67.3654	0.7715	0.0018	0.0015	67.9874
ADPO-LSTM	0.9705	0.0008	0.0005	29.7854	0.9615	0.0012	0.0005	32.1478

**Table 14:** The average performance across all stations during the training phase.

	R^2^	RMSE	MAE	COV
LSTM	0.6443	0.0026	0.0020	99.8489
PO-LSTM	0.8293	0.0014	0.0012	59.6033
SCA-LSTM	0.8233	0.0016	0.0013	62.0916
WOA-LSTM	0.8113	0.0018	0.0015	66.2705
SOA-LSTM	0.8193	0.0016	0.0014	63.7243
HHO-LSTM	0.8320	0.0014	0.0011	58.6241
ADPO-LSTM	0.9792	0.0003	0.0002	24.0902

**Table 15:** The average performance across all stations during the testing phase.

	R^2^	RMSE	MAE	COV
LSTM	0.6125	0.0037	0.0031	104.2516
PO-LSTM	0.7998	0.0022	0.0017	63.5242
SCA-LSTM	0.7710	0.0023	0.0019	66.0622
WOA-LSTM	0.7465	0.0026	0.0021	70.0438
SOA-LSTM	0.7610	0.0024	0.0020	67.6685
HHO-LSTM	0.8008	0.0021	0.0016	62.7754
ADPO-LSTM	0.9726	0.0010	0.0006	27.7271

**Table 16:** Comparison with other optimized DNN approaches through the testing phase for stations A and B.

	Station A				Station B	
	R^2^	RMSE	MAE	R^2^	RMSE	MAE
ADPO-LSTM	0.9785	0.0009	0.0008	0.9798	0.0010	0.0005
ADPO-Bi-LSTM	0.8895	0.0014	0.0013	0.8985	0.0015	0.0013
ADPO-KELM	0.8625	0.0014	0.0012	0.9125	0.0020	0.0012
ADPO-ELM	0.7945	0.0021	0.0018	0.8285	0.0019	0.0015
ADPO-RF	0.8075	0.0019	0.0016	0.8495	0.0017	0.0014

**Table 17:** Comparison with other optimized DNN approaches through the testing phase for stations C and D.

	Station C				Station D	
	R^2^	RMSE	MAE	R^2^	RMSE	MAE
ADPO-LSTM	0.9705	0.0008	0.0005	0.9615	0.0012	0.0005
ADPO-Bi-LSTM	0.9205	0.0016	0.0008	0.9185	0.0010	0.0008
ADPO-KELM	0.9025	0.0021	0.0018	0.9055	0.0011	0.0008
ADPO-ELM	0.8175	0.0024	0.0020	0.8385	0.0017	0.0014
ADPO-RF	0.8375	0.0022	0.0019	0.8325	0.0016	0.0013

**Table 18:** The average metrics of various DNN approaches.

	R^2^	RMSE	MAE
ADPO-LSTM	0.9726	0.0010	0.0006
ADPO-Bi-LSTM	0.9068	0.0014	0.0011
ADPO-KELM	0.8958	0.0017	0.0013
ADPO-ELM	0.8198	0.0020	0.0017
ADPO-RF	0.8318	0.0019	0.0016

**Table 19:** Average metrics with other state-of-the-art approaches.

	R^2^	RMSE	MAE
LSTM + HBO [67]	0.9654	0.042869	0.02998
RVFL + CapSA [68]	0.9681	110.3154	64.452775
RVFL + SCA [68]	0.9562	128.4209	71.53655
RVFL + GWO [68]	0.9374	154.2171	102.3056
ADPO-LSTM (Proposed)	0.9726	0.001010	0.00060

**Table 20:** Wilcoxon test *p*-values for ADPO-LSTM versus other approaches.

	Station A	Station B	Station C	Station D
ADPO-LSTM vs. PO-LSTM	0.0012	0.0008	0.0015	0.0011
ADPO-LSTM vs. SCA-LSTM	0.0007	0.0006	0.0009	0.0008
ADPO-LSTM vs. WOA-LSTM	0.0003	0.0004	0.0005	0.0006
ADPO-LSTM vs. SOA-LSTM	0.0005	0.0007	0.0008	0.0009
ADPO-LSTM vs. HHO-LSTM	0.0018	0.0021	0.0019	0.0017
ADPO-LSTM vs. LSTM	<0.0001	<0.0001	<0.0001	<0.0001

## Data Availability

The data presented in this study are available on request from the corresponding author.
